# The quality of clinical practice guidelines for preoperative care using the AGREE II instrument: a systematic review

**DOI:** 10.1186/s13643-020-01404-8

**Published:** 2020-07-13

**Authors:** Agustín Ciapponi, Elena Tapia-López, Sacha Virgilio, Ariel Bardach

**Affiliations:** Instituto de Efectividad Clínica y Sanitaria (IECS-CONICET), Emilio Ravignani 2024 (C1414CPV), Buenos Aires, Argentina

**Keywords:** Perioperative care, Clinical practice guidelines, Systematic review, GRADE, AGREE-II

## Abstract

**Background:**

Our aim was to summarize and compare relevant recommendations from evidence-based CPGs (EB-CPGs).

**Methods:**

Systematic review of clinical practice guidelines. Data sources: PubMed, EMBase, Cochrane Library, LILACS, Tripdatabase, and additional sources. In July 2017, we searched CPGs that were published in the last 10 years, without language restrictions, in electronic databases, and also searched specific CPG sources, reference lists, and consulted experts. Pairs of independent reviewers selected EB-CPGs and rated their methodological quality using the AGREE-II instrument. We summarized recommendations, its supporting evidence, and strength of recommendations according to the GRADE methodology.

**Results:**

We included 16 EB-CPGs out of 2262 references identified. Only nine of them had searches within the last 5 years and seven used GRADE. The median (percentile 25–75) AGREE-II scores for rigor of development was 49% (35–76%) and the domain “applicability” obtained the worst score 16% (9–31%). We summarized 31 risk stratification recommendations, 21.6% of which were supported by high/moderate quality of evidence (41% of them were strong recommendations), and 16 therapeutic/preventive recommendations, 59% of which were supported by high/moderate quality of evidence (75.7% strong). We found inconsistency in ratings of evidence level. “Guidelines’ applicability” and “monitoring” were the most deficient domains. Only half of the EB-CPGs were updated in the past 5 years.

**Conclusions:**

We present many strong recommendations that are ready to be considered for implementation as well as others to be interrupted, and we reveal opportunities to improve guidelines’ quality.

## Implication statement

We identified many risk stratification and therapeutic strong recommendations that can be implemented and other ones usually followed by many anesthesiologists in their daily practice that should be interrupted.

Finally, we described opportunities to improve guidelines’ quality.

## Background

An estimated 313 million major surgical procedures are undertaken every year worldwide [1]. Low- and high-income countries show an estimated rate of major surgery of 295 and 11,110 procedures per 100,000 population per year respectively [1], an enormous disparity for the recommended minimum threshold of 5000 operations per 100,000 people, that is associated with desirable health outcomes. At current rates of surgical and population growth, 6.2 billion people (73% of the world’s population) will be living in countries below the minimum recommended rate of surgical care in 2035 [2]. However, the crude number of patients who receive surgery is increasing, as well as their mean age and the occurrence of comorbidities [3]. Because of the inherent risks of death and complications, surgical safety is a significant public-health concern. As examples, 2.4% (95% CI 2.1 to 2.6%) of patients undergoing surgery will suffer major cardiac complications [4], and 5% (95% CI 4.5 to 5.5%) will have a perioperative myocardial infarction [5]. In this context, to provide adequate preoperative care is truly mandatory. The first routine preoperative tests started 50 years ago with only a handful of actions and have nowadays expanded to a large set of risk stratification or diagnostic tests to define the preoperative clinical risk categories and also many preventive interventions. Lately, efforts to standardize care have been made, specially through the implementation of clinical practice guidelines (CPGs) with recommendations useful both for health providers and patients [6]. These recommendations usually consider all risks and benefits for a risk stratification or therapeutic procedure to be undertaken, sometimes even including algorithm pathways. The potential benefits, like the safety of care and standardization of procedures, are only as good as the quality of the practice guidelines implemented. Unfortunately, those CPGs not supported by the best evidence might promote inappropriate preoperative testing behaviors, negative both for patients and health systems. For example, false positive results, coming from inappropriate testing, may delay or prevent surgery, thus creating unnecessary stress or harm to patients.

Multiple medical societies and organizations around the world have published preoperative evaluation CPGs; however, many of them are not even based on solid scientific evidence. Additionally, not all of them harness methods like the Grading of Recommendations Assessment, Development, and Evaluation (GRADE) approach, which is one of the soundest system for rating the quality of a body of evidence in systematic reviews and CPGs [7]. GRADE offers a transparent and structured process for developing and presenting evidence summaries and making recommendations [7].

A systematic review found no evidence from high quality studies to support routine preoperative tests in healthy adults undergoing non-cardiac surgery [8]. Risk stratification testing based on the problems identified during the preoperative assessment seems justified, but there is still little evidence supporting it [8]. In this way, the implementation of EB-CPGs may lead to a reduction in the number of unnecessary preoperative tests, without affecting patient safety [9–13]. The first health technology assessment (HTA) on the topic published in 1989 by the Swedish Council on Technology Assessment in Health Care (SBU) [14], showed healthcare quality improvements and cost savings using an evidence-based approach. The findings of this report have been confirmed by nine other subsequent studies from five countries, collected in another HTA document [15].

For this reason, through an overview of clinical practice guidelines, we aimed to identify and synthetize EB-CPGs on preoperative care that were published worldwide in the last 10 years, in order to help prioritization processes. We also rated CPGs’ quality and summarized recommendations describing their level of evidence and the strength of recommendations according to the GRADE approach [7].

## Methods

### Study design

We performed a systematic review (overview) of EB-CPGs following Cochrane methods [16] and the Argentinean Academy of Medicine’s Guide for the adaptation of CPGs for searching and selecting CPGs [17]. For reporting, we followed the PRISMA statement [18] and a specific guideline for overviews of systematic reviews (Online supplemental material. Appendix 1. PRISMA checklist) [19]. The protocol is available in Spanish including a summary in English.^1^

We aimed to identify the most reliable CPGs; therefore, we used a definition for EB-CPGs previously reported [20]. The inclusion eligibility criteria (all criteria required) were as follows:

- CPGs of perioperative care published in the last 10 years including those recommendations potentially applicable to any kind of surgery, not site or condition-specific
- b) Provides a list of the CPG development panel members including their expertise or qualifications.
- Use standard methods such as Cochrane methods, Equator Network-proposed checklists, or any sufficiently detailed method allowing reproducibility of the identification, data collection, and study risk of bias assessment.
- Report of the level of evidence that supports each recommendation

Exclusion criteria (any criterion required) were as follows:

- Guidelines limited to single specific conditions such as obesity, renal disorders, or pheochromocytoma
- Guidelines limited to single specific body part surgeries such as neurosurgery or colorectal surgery.
- Guidelines including some recommendations but whose entire focus was clearly not the preoperative care

### Search strategy

In July 2017, we searched CPGs published in the last 10 years without language limitations in main electronic databases, metasearch engines, specific CPG sources, reference lists and consultation of experts, and the main scientific societies related to preoperative evaluation. The sources included PubMed, EMBase, Cochrane Library, LILACS, Tripdatabase, and additional sources: National Guideline Clearinghouse, NeLH Guidelines Finder, Guía Salud GPCs en España, GAC guidelines, CMA Infobase: Clinical Practice Guidelines Database (CPGs), New Zealand Guidelines, Scottish Clinical Guidelines, EBM Guidelines, Health Services/Technology Assessment Text (HSTAT), National Institute for Health and Clinical Excellence (NICE), and Institute for Clinical Systems Improvement (ICSI). See Online supplemental material. Appendix 2. Search strategy for details of these sources and our search strategy for preoperative care. The search strategy was developed by a trained librarian, and the citations were initially managed for deduplication trough EndNote 9® reference manager.

### Selection and data extraction

Pair of reviewers independently selected (by title and abstract first, and full text eligible studies afterwards) the articles retrieved, with a specific software to facilitate the initial phases of systematic reviews called Early Review Organizing Software (EROS) [21]. One reviewer extracted them while the other verified the data in a previously piloted form (which included variables such as search date, objective, setting, target population, target professionals, recommendations, classification system of the quality of evidence and of the strength of the recommendation, quality of evidence by recommendation, and the strength of each recommendation) and preoperative clinical risk criteria and categories (see Online supplemental material 3. Preoperative clinical risk criteria and categories). Discrepancies were resolved by a consensus of the whole team.

### Guideline quality appraisal and classification

Independent pairs of reviewers rated each EB-CPGs using the AGREE-II tool consisting of 23 key items organized in six domains: scope and purpose, stakeholder involvement, rigor of development, clarity of presentation, applicability, editorial independence, and two overall evaluation items [22]. Each item was graded using a scale of 7 points: from 1, meaning “Strongly disagree,” to 7, meaning “Strongly agree.” The total was presented as a percentage of the maximum possible score for that domain (from 0 to 100%). We present the AGREE-II domain scores expressed as a percentage across CPGs (Online Supplemental material. Appendix 5 with the explanation of each items of the AGREE-II domains). Discrepancies were resolved by a consensus of the whole team.

We also categorized each EB-CPGs according to the extent to which they successfully addressed AGREE-II criteria as follows [17]:

- Strongly recommended (++), CPG whose standardized score exceeds 60% in ≥ 4 AGREE-II domains. The scores of the remaining domains must be ≥ 30% and > 60% for the domain rigor of development.
- Recommended (+), CPG whose standardized score ranges from 30 to 60% in ≥ 4 AGREE-II domains. The rigor of development score must be between 30 and 60%.
- Not recommended (–), CPG whose standardized score is < 30% in ≥ 4 AGREE-II domains or if rigor of development score is less than 30%.

To deal with discrepancies between the direction and strength of the CPG recommendations, we applied a rule to decide “doing or not doing the recommendation”:

- Yes (Y)–no (N) to doing it: ≥ 2/3 recommendations in the same direction (for/against) and ≥ 2/3 strong recommendations.
- Probably yes (PY)–probably no (PN) to doing it: ≥ 2/3 recommendations in the same direction (for/against) and < 2/3 strong recommendations.
- Uncertainty (?) to do it: < 2/3 recommendations in the same direction (for/against).

### Synthesis of results

We conducted a tabular synthesis of the recommendations to describe their strength and the level of evidence supporting them according to the current GRADE methodology [7], and transforming the original grading system when necessary, to compare and integrate the results for each recommendation in a unified manner. Simply put, the GRADE quality of evidence can be HIGH, MODERATE, LOW and VERY LOW. The Randomized Clinical Trials (RCTs) start from HIGH quality of evidence, and the non-randomized studies start from a LOW quality of evidence. Five criteria can downgrade one or two levels: methodological quality (study limitations), inconsistency of results, indirectness, imprecision, and publication bias. In cases where there are no methodological limitations, there are three criteria that can upgrade one or two levels: magnitude of effect, dose-response effect, and confounders underestimating the effect. For mapping the level of evidence to a common grading system (GRADE), we re-assessed all evidence when the translation was not obvious. Pair of reviewers independently extracted or reassessed the level of evidence, and discrepancies were resolved by a consensus of the whole team. Regarding the strength of a recommendation, which is defined as the extent to which one can be confident that the desirable consequences of an intervention outweigh its undesirable consequences, GRADE uses four simple categories to classify them. The categories are “strong” or “weak” and “for” or “against” a certain risk stratification or therapeutic approach. We presented descriptive statistics as percentages or means with standard deviations.

## Results

### Search results

**Fig. 1:**
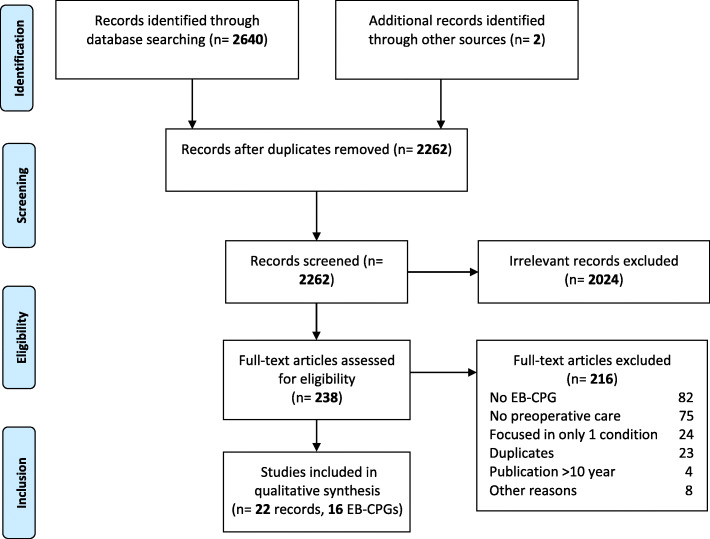
Study flowchart

The search strategy identified 2262 references after the elimination of duplicates. After the selection process, we identified 23 references corresponding to 16 EB-CPGs published in the last 10 years (Fig. 1 flowchart). Two references were examined in depth and eventually excluded since they only transcribed pre-existing CPGs, already included in our selection [23, 24].

### Guideline characteristics

Table 1 provides a general description of the included EB-CPGs. Seven were developed in America (4 in the USA, 1 in Argentina, 1 in Brazil, and 1 in Canada), seven in Europe (2 continental, 2 from Italy, 1 in Belgium, 1 in Scotland, and 1 in UK), and two were global collaborations. Only 8/16 (50%) of the EB-CPGs that reported their search date, conducted their searches within the last 5 years. Out of the EB-CPGs, ten addressed multiple practices, five focused on unique practices, one referred to perioperative fasting, and the remaining four were about antimicrobial prophylaxis. Furthermore, four were risk stratification recommendations, five were therapeutic or preventive interventions, and six considered both aspects.

**Table 1:** General description of the EB-CPGs included

Development entity			Literature search year	Title	Single or multiple practice	Type of practice evaluated*	^**§**^Guide Quality 1 = lowest 7 = highest
Acronym—guide publication year	Full name	Location					
NICE 2016 [25]	National Institute of Health and Care of Excellence	UK	2015	Clinical Guidelines. Preoperative tests (update): routine preoperative tests for elective surgery	**6**	Dx	**6**
ESC/ESA 2014 [26]	European Society of Cardiology/European Society of Anesthesia	Europe^†^	2014	ESC/ESA Guidelines on non-cardiac surgery: cardiovascular assessment and management	**4.5**	Dx, Tx	**4.5**
SBC 2017 [27]	Brazilian Society of Cardiology	Brazil	2016	3rd guide for the perioperative evaluation of the Brazilian Society of Cardiology	**4.5**	Dx, Tx	**4.5**
ACC/AHA 2014 [28]	American College of Cardiology/American Heart Association	USA	2013	ACC/AHA guideline on perioperative cardiovascular evaluation and management of patients undergoing noncardiac surgery	**6**	Dx	**6**
CCSG 2017 [29]	Canadian Cardiovascular Society	**Canada**	2015	Guidelines on perioperative cardiac risk assessment and management for patients who undergo noncardiac surgery	**3**	Dx, Tx	**3**
ERAS Society 2012 [30]	Society of post-surgical recovery	World	2012	Guidelines for perioperative care in elective rectal/pelvic surgery: enhanced recovery after surgery	**4**	Dx, Tx	**4**
SARNePI 2014 [31]	Italian Society of Anesthesia and Intensive Pediatric Therapy and Neonatology	Italy	2012	Preoperative evaluation in infants and children: recommendations of the Italian Society of Pediatric and Neonatal Anesthesia and Intensive Care (SARNePI).	**4**	Dx	**4**
ICSI 2012 [32]	Institute for the Improvement of Clinical Systems	USA	2012	Pre-operative evaluation	**4**	Dx, Tx	**4**
ERAS Society 2016 [33]	Post-Surgical Recovery Society	World	2014	Guidelines for pre- and intra-operative care in gynecologic/oncology surgery: enhanced recovery after surgery	**4**	Dx, Tx	**4**
ESA 2011 [34]	European Society of Anesthesia	Europe^†^	2009	Perioperative fasting guide in adults and children	**4**	Tx	**4**
BARA 2013 [35]	Regional Anesthesia Associations of Belgium	Belgium	NR	Recommendations and guidelines for obstetric anesthesia in Belgium	**3**	Dx	**3**
ASHP 2013 [36]	American Society of Health-System Pharmacists	USA	2010	Clinical practice guidelines for antimicrobial prophylaxis in surgery	**5.5**	Tx	**5.5**
SIGN 2014 [37]	Scottish Intercollegiate Guidelines Network	Scotland	2007	Antimicrobial prophylaxis in surgery	**6.5**	Tx	**6.5**
CDC 2017 [38]	Center for Disease Control	USA	2014	Guideline for the prevention of surgical site infections	**6**	Tx	**6**
PNLG 2009 [39]	National Program of Italian Guides	Italy	NR	Perioperative antibiotic prophylaxis in adults	**3**	Tx	**3**
SAC 2016 [40]	Argentine Society of Cardiology	Argentina	2015	Argentine Consensus on Cardiovascular Risk Assessment in Non-Cardiac Surgery	**6**	Dx, Tx	**6**

*NR* not reported

*Diagnostic practice (Dx), therapeutic/preventive (Tx)

^**§**^Based on AGREE-II tool. See supplemental materials for more details

^†^The whole continent

Each guideline reports the levels of evidence and the recommendation grading systems used by their authors. The grading system used were GRADE (7 EB-CPGs), SIGN [41] (2 EB-CPGs), and the others utilized their own or modified systems (Online supplemental material Appendix 4)**.**

We presented the scores as a percentage per each AGREE-II domain. The domains with the best median score (percentile 25–75) were editorial independence 91% (81–100), clarity of presentation 85% (69–97), and scope and objective 80% (65–89). Stakeholder involvement 53% (44–62) and rigor of development 49% (35–76) had an intermediate performance while “applicability” was the most deficient 16% (9–31). Regarding the guideline recommendation category, 6/16 (37%) were classified as highly recommended and the rest as recommended (Online supplemental material Appendix 5). An overall AGREE-II score is also presented in Table 1.

### Risk stratification recommendations (diagnostic tests)

Table 2 shows the risk stratification recommendations presenting the level of evidence and recommendation strength of the EB-CPG with the highest overall and methodological rigor AGREE-II score. The 31 risk stratification recommendations included 102 specific recommendations according to the population/problem or the type of surgery. Out of the 102 recommendations, 5 (4.9%) had a high level of evidence, 17 (16.7%) had a moderate level, 45 (44.1%) had a low level, and 35 (34.3%) had a very low/insufficient level. Regarding recommendation strength, 24 (23.5%) were strong for, 26 (25.5%) were strong against, 37 (36.3%) weak for, and 15 (14.7%) were weak against.

**Table 2:** Risk stratification, GRADE level of evidence and strength of recommendation by clinical specialties

**General requirements**		
1. **Preoperative evaluation**		
Pediatric patients receiving anesthesia	Very low	Strong for
Emergency surgeries in pediatric patients	Very low	Strong against
All patients who are undergoing diagnostic or therapeutic procedures	Very low	Weak for
Patients with ASA 1 or 2 without surgical or obstetric history (preanesthetic evaluation, including physical examination, the day of the procedure).	Very low	Weak for
Patient with significant medical, surgical, or obstetrical history (anesthesiologist assessment)	Very low	Weak for
In case of bleeding or complication history of previous alloimmunization, it is recommended to evaluate the blood type.	Very low	Weak for
2. **Informed consent** (Ideally written)		
Provide information on risks and benefits related to obstetric anesthesia and analgesia.	Very low	Weak for
3. **Complete laboratory**		
Patients undergoing low-risk surgery independently of their ASA score	Very low	Strong against
Patients undergoing intermediate-risk surgery	Very low	Strong against
Patients with renal or cardiovascular disease undergoing intermediate-risk surgery that has not been recently evaluated	Very low	Weak for
Patients undergoing high-risk surgery	Very low	Strong for
Patients with preeclampsia or other preceding or a suspect of hemostatic disorder, it is recommended to apply platelet count, liver function test, and evaluation of coagulation	Very low	Weak for
In case of bleeding or complication history of previous alloimmunization, it is recommended to evaluate the blood type.	Very low	Weak for
Patients with liver failure	Very low	Strong for
In anticoagulated patients (e.g., consume Warfarin)	Low	Strong for
Patients with potential risk of bleeding undergoing intermediate or high-risk surgery	Very low	Strong for
Routinely	Very low	Strong against
4. **Hematocrit and hemoglobin**		
In pediatric patients with possible bleeding	Low	Strong for
In pediatric patients routinely perform minor surgery	Low	Strong against
Patients with anemia or blood disease or liver disease; when you suspected of anemia or other chronic disease during clinical examination. In medium or high-risk surgeries, anticipated transfusion requirement	Low	Strong for
Patients requiring intermediate or major surgery, and bleeding risk of transfusion requirement	Low	Strong for
Patients over 40 years	Low	Weak for
Patients with a history of hematological or liver disease	Low	Strong for
5. **Hemostasis/coagulation tests**		
Pediatric patients with negative history	Low	Strong against
Patients with a history of bleeding	Low	Strong for
Patients with liver failure	Very low	Strong for
In anticoagulated patients (e.g., consume Warfarin)	Low	Strong for
Patients with potential risk of bleeding undergoing intermediate or high-risk surgery	Very low	Strong for
Routinely	Very low	Strong against
6. **Urinalysis**		
Routinely before surgery	Very low	Weak against
Urine or culture if diagnosing a urinary infection can influence surgery decisions	Very low	Weak for
7. **Glucose**		
Routinely to pediatric patients	Low	Strong against
Diabetic patients	Low	Strong for
8. **Glycated hemoglobin (HbA1c) test**		
Diabetic patient without Hb1Ac within 3 months	Very low	Weak for
Patients without diabetes	Very low	Weak against
9. **Assessment of risk factors for surgical site infection**		
Assessment of smoking, diabetes, obesity, malnutrition, and chronic skin disease	Low	Strong for
10. **Kidney function tests**		
For minor surgery in ASA 1/2 patients or intermediate-risk surgery in ASA 2 patients	Very low	Weak against
For complex or major surgery in ASA 1 patients at risk of acute kidney injury (AKI)	Very low	Weak for
In intermediate-risk surgery in ASA 2 patients at risk of AKI. In patients with increased risk surgery performed	Very low	Weak for
ASA 3/4 patients: at risk of AKI in low-risk surgery or just higher-risk surgery	Very low	Weak for
11. **Sickle cell disease/trait test**		
Routinely	Very low	Weak against
Assess personal of family history of sickle cell anemia	Very low	Weak against
Contact a specialized service providing treatment to a confirmed case	Very low	Weak for
12. **Chest X-ray**		
Routinely in healthy people	Low	Strong against
Patients with a history or diagnostic tests suggesting cardiorespiratory disease	Moderate	Weak for
Patients over 40 years, patients undergoing non-low-risk surgery	Low	Weak for
Patients undergoing non-low-risk surgery or mainly intrathoracic or intraabdominal surgery	Moderate	Weak for
13. **Pregnancy testing**		
Performed in women of childbearing age	Very low	Weak for
Test the day of surgery in women of childbearing age. In pregnant women, ensure that surgery and anesthesia does not threaten the fetus life. Document all discussions with women about whether to carry out a pregnancy test. Carry out the pregnancy test under the possibility of pregnancy.	Very low	Strong for
**Cardiovascular requirements**		
14. **Electrocardiography:**		
In neonates and/or children of 6 months	Low	Weak for
Healthy people undergoing minor surgery	Low	Strong against
Perform in cases of clinical suspicion	Low	Weak for
People over 65 undergoing minor or intermediate surgery	Very low	Strong against
People with cardiovascular disease	Low	Weak for
People with a morbidity undergoing intermediate or major surgery	High	Strong for
15. **Effort electrocardiography**		
Patients undergoing surgeries of intermediate or high risk of complications, including arterial vascular surgery (without severe cardiovascular perioperative conditions)	Low	Weak for
Patients undergoing low-risk surgery	Low	Strong against
Patients undergoing intermediate-risk surgery	Low	Strong against
16. **Resting echocardiography**		
**High-risk surgery**		
Patient with suspected moderate or severe valvular involvement without evaluation in the last year or with worsening of symptoms	Low	Strong for
Patient with heart failure or symptoms suggestive of heart problems, without assessment in the past year, undergoing cardiac surgery	Low	Weak for
Symptomatic patients with stent grafts who go to surgery and who have no evaluation in the last year	Low	Strong for
Asymptomatic patients	Low	Weak for
**Low, intermediate or uncertain surgical risk**		
Routine test in asymptomatic patients without suspect of heart failure or severe valvular disease	Very low	Weak against
17. **Effort echocardiography**		
Routinely to assess cardiac risk	Low	Strong against
18. **Tomographic coronary angiography**		
Routinely to assess cardiac risk	Moderate	Strong against
19. **Assessment of left ventricular function**		
Patients suspected to have valvular disease with important clinical manifestations or undergoing liver transplantation	Low	Weak for
Patients with heart failure without ventricular function assessment	Low	Weak against
Patients undergoing high-risk surgery	Moderate	Weak for
Obese patients (BMI ≥ 40) undergoing bariatric surgery	Low	Weak for
Routinely	Moderate	Strong against
20. **Natriuretic peptide**		
Patients undergoing cardiac surgery	High	Weak for
Patients over 55 years with at least one cardiovascular risk factor undergoing non-cardiac surgery	Low	Weak for
21. **Brain natriuretic peptide (BNP) or NT-proBNP**		
Patients over 65 years or patients between 45 and 64 years with significant cardiovascular disease or score (revised cardiac risk index (RCRI) ≥ 1	Moderate	Strong for
22. **Troponin**		
Troponin prior to vascular surgery	Moderate	Weak for
Troponin as a preoperative marker of cardiovascular risk and mortality in non-cardiac surgery	Low	Weak for
23. **Coronary angiography**		
The indications of angiography and coronary revascularization are those of non-surgical context	Moderate	Strong for
Urgent angiography in patients with myocardial infarction without ST elevation requiring elective non-cardiac surgery or with a computed tomography (CT) with multiple cuts showing serious injury of the left coronary trunk	Low	Weak for
Urgent or early invasive strategy for patients with NSTEMI requiring elective non-cardiac surgery	High	Strong for
Patients with recent coronary disease at high clinical risk, functional class III-IV in the last 6 months, or patients with severe valve disease and concomitant coronary heart disease	Low	Strong for
Patients with non-high-risk criteria (**Annex 5**) and functional or pharmacological stress tests showing myocardial ischemia	Low	Weak against
Patients with or without stable coronary disease functional class I-II without evidence of ischemia by stress tests, or those with severe coronary disease according CT multislice (excluding injury of left coronary trunk) clinically stable without ischemia, or in patients whose non-cardiac surgery cannot be delayed more than 2 weeks due to the underlying disease	Low	Strong against
24. **Noninvasive test for myocardial ischemia**		
Patients undergoing intermediate or high-risk surgery (without severe cardiovascular perioperative conditions) and those undergoing arterial vascular surgery	Moderate	Weak for
Intermediate or high-risk patients with poor functional capacity undergoing intermediate-risk surgery	Moderate	Weak against
Patients undergoing low-risk surgery	Low	Strong against
Low-risk patients undergoing low or intermediate-risk surgery	Low	Strong against
**Pulmonary requirements**		
25. **Polysomnography**		
In patients requiring continuous positive airway pressure (CPAP)	High	Strong for
Patients presumed to have obstructive sleep apnea (OSA) based on the preoperative history and physical examination	Low	Weak for
26. **Lung function tests**		
Spirometry in patients undergoing non-high-risk surgery	Very low	Strong against
Arterial blood gas analysis in patients undergoing non-high-risk surgery	Very low	Strong against
Assessment by medical senior anesthesiologist after confirming respiratory illness or suspected in patients ASA 3/4 undergoing high-risk surgery	Very low	Weak for
**High risk surgery requirements**		
27. **Stress testing**		
In high-risk patients with unknown functional capacity	Moderate	Weak against
Patients with major criteria of high cardiovascular risk (**Annex 5**)	Low	Strong against
For high-risk patients and moderate to good (≥ 4 METs to 10 METs) functional capacity	Low	Weak against
For high-risk patients and poor (< 4 METs) or unknown functional capacity, if it will change management.	Low	Weak against
Patients with low risk and a poor (< 4METs) or unknown functional capacity, who have angina or dyspnea functional class I-II	Low	Weak for
Patients with low clinical risk criteria established in **Annex 5**, who are asymptomatic and with good functional class	Low	Weak against
Routinely for patients undergoing low-risk noncardiac surgery	Moderate	Strong against
28. **Stress test image**		
For high-risk surgery patients with two or more clinical risk factors and low functional capacity	Low	Strong for
For intermediate and high-risk patients with one or two clinical risk factors and poor functional capacity (< 4MET)	Very Low	Weak against
For low-risk patients regardless of the clinical state of patient	Very low	Strong against
**Special situations or considerations**		
29. **Cardiopulmonary stress test**		
Cardiopulmonary exercise testing to improve the estimation of cardiac risk	Low	Strong against
High-risk patients with unknown functional capacity	Moderate	Weak against
30. **Pharmacological stress test**		
Patients undergoing non-cardiac surgery who have poor functional capacity (< 4 METS) dobutamine stress test	Moderate	Weak for
Routinely in asymptomatic patients who are at low-risk surgery	Moderate	Strong against
31. **Prokinetic and other interventions**		
Routine use of antacids, metoclopramide, or H2-receptor antagonists before elective surgery in non-obstetric patients	High	Strong against
H2-receptor antagonists the night before and the morning of elective cesarean section	Moderate	Strong for
Intravenous H2-receptor antagonist before emergency cesarean section; supplemented with 30 ml of sodium citrate if general anesthesia is planned	Moderate	Strong for

The presented level of evidence and recommendation strength comes from the EB-CPG with the highest overall and methodological rigor AGREE-II score. The level of evidence and recommendation strength by EB-CPG is presented in the online supplemental material 6.a

We found discrepancies among EB-CPG in 10 out of 102 (10%) risk stratification recommendations After applying the rule “doing or not doing the recommendation,” 31 (60 specific) are “doing” and 31 are (39 specific) “not doing” diagnostic evaluations (see Online supplemental material Appendix 6. Table 1 GRADE level of evidence and strength of recommendations by CPG; Table 2 Recommended risk stratification evaluations only and Table 3 Not recommended risk stratification evaluations to facilitate the finding of relevant recommendation by different point of access).

### Therapeutic/preventive recommendations

Table 3 shows the therapeutic/preventive recommendations using the same presenting criterion in Table 2. The 16 therapeutic/preventive recommendations included 78 specific recommendations according to the population or the type of surgery. Out of these recommendations, there were 28 (35.9%) with a high evidence level, 18 (23.1%) with a moderate level, 24 (30.8%) with a low level, and 8 (10.2%) with a very low level. Regarding their recommendation strengths, 41 (52.6%) were strong for, 18 (23.1%) were strong against, 14 (17.9%) weak for, and 5 (6.4%) weak against. In Online supplemental material Appendix 7, we present an additional table concerning antimicrobial prophylaxis recommendations for each surgical site.

**Table 3:** Therapeutic/preventive care, GRADE level of evidence and strength of recommendation*

Recommendation	Level of evidence	Strength of recommendation
**General recommendations**		
1. **Smoking cessation**		
Smoking cessation advice	Low	Strong for
2. **Fast**		
Stop fluid intake in children and adults at least 2 h before elective surgery in	Moderate	Strong for
Stop intake of solids in children and adults 6 h before surgery	Moderate	
Stop intake in infants up to 4 h before surgery and 6 h in those who consume other milk	Low	
Intake of clear fluids (including water, clear juice, and tea or coffee without milk) in children and adults up to 2 h before elective surgery.	Moderate	
3. **Carbohydrate intake**		
Intake until 2 h before surgery in nondiabetics	Moderate	Strong for
Taking high carbohydrate drinks to 2 h before elective surgery even in diabetic patients	High	
Drinking liquids rich in carbohydrates before elective surgery improves subjective well-being, reduces thirst and hunger and reduces postoperative insulin resistance	High	
4. **Alcohol intake**		
Avoid drinking 4 weeks before, especially in rectal surgery.	Moderate	Strong for
5. **Bowel preparation (cleansing)**		
With or without planned bowel resection	Moderate	Strong against
6. **Antimicrobial prophylaxis** (see **Annex 2** for specific antibiotic recommendation details)		
Antibiotics intravenous (first generation cephalosporin or amoxicillin/clavulanate) routinely 60 min before the incision. Further doses for prolonged surgery, severe blood losses and obese patients	Low	Weak for
Vancomycin monotherapy	Low	Weak against
For insertion of a pacemaker or cardiac defibrillator, in open surgery including coronary bypass and valve prosthesis placement	High	Strong for
For lung resection	Moderate	Strong for
For clean-contaminated head and neck surgery	High	Strong for
For adenotonsillectomy	High	Weak against
For ear surgery including myringoplasty	High	Strong against
For nasal and paranasal sinus surgeries	Moderate	Strong against
For clean head and neck surgery	Very low	Strong against
For colorectal surgery	High	Strong for
For oncological breast surgery and reduction mammoplasty	High	Strong for
For endoscopic gastrostomy and stomach and duodenum surgery	Moderate	Strong for
For clean-contaminated procedures esophagus and small intestine	Very low	Weak for
For appendectomy, open biliary surgery, liver resection surgery, pancreatic surgery, breast augmentation	High	Strong for
For inguinal hernia repair with or without use of prosthetic material, laparoscopic hernia surgery with or without prosthetic material, diagnostic laparoscopy and excisional lymph node biopsy	High	Strong against
For laparoscopic cholecystectomy surgery	High	Strong against
Intranasal mupirocin in adult patients undergoing surgery with a high risk of major morbidity due to *S. aureus* or MRSA	High	Strong for
For craniotomy and cerebrospinal flow deviation	High	Strong for
For induction of abortion and cesarean section	High	Strong for
For abdominal and vaginal hysterectomy	Moderate	Strong for
For salpingo-oophorectomy and ovarian tissue excision or reconstruction	High	Strong against
For ankle prosthesis implantation	High	Strong for
For knee prosthesis implantation	Low	Strong for
For closed fracture fixation, mounting a prosthetic device when there is no direct evidence available, ankle fracture repair	High	Strong for
For spinal surgery	Moderate	Strong for
For elective orthopedic surgeries without use of prosthesis	Very low	Strong against
For transurethral resection of the prostate, lithotripsy	High	Strong for
For transrectal prostate biopsy, radical prostatectomy, radical cystectomy, surgery of renal parenchyma, nephrectomy and removal of hydrocele	Moderate	Strong for
For transurethral resection of bladder tumors	Very low	Strong against
For lower limb amputation and arterial surgery in the abdomen or lower extremities	Moderate	Strong for
For carotidal thromboendarterectomy, endarterectomy, tubal surgery varicose veins and other venous occlusions	Very low	Strong against
Antibiotic must have a spectrum of action against likely contaminants	Very low	Weak for
Avoid beta-lactam antibiotics in patients with a history of anaphylaxis, urticaria, or rash appearing immediately after treatment with penicillin	Low	Weak for
Antibiotic prophylaxis should begin immediately before anesthesia and, in any case, of 30 to 60 min before the first skin incision	High	Strong for
More than single antibiotic dose (except in special situations)	Very low	Strong against
Additional intraoperative dose of antibiotic in adults, to be held after the fluid replenishment, if a loss of more than 1500 ml of blood is verified during the operation or after hemodilution of more 15 ml per kg	Very low	Weak for
Consider the increased risk clostridium difficile infection associated with some antibiotics like cephalosporins, clindamycin, fluoroquinolones, carbapenems	Low	Weak for
Consider glycopeptides for prophylaxis in patients undergoing high-risk surgery that are positive for MRSA	High	Strong for
Registering a minimum set of data on medical history and treatment forms to assess the suitability of perioperative antibiotic prophylaxis	Very low	Strong for
7. **Preanesthetic medication**		
Benzodiazepines	Moderate	Weak against
8. **Thromboprophylaxis**		
Compression stockings	High	Strong for
Low molecular weight heparin		
Continuation of contraceptives		
9. **Surgical site preparation**		
Alcohol-chlorhexidine use	High	Strong for
Antimicrobial agents (i.e., ointments, solutions, or powders) for prevention of surgical site infection	Low	Strong against
Hair clipping	High	Strong for
Adhesive strips of plastic with or without antimicrobial properties	Moderate	Weak against
Microbial sealant after intraoperative skin preparation	Low	Weak against
Patients bath with antiseptic agent at least one night before surgery	Moderate	Strong for
10. **Prokinetic**		
For obstetrical patients	Moderate	Strong for
For non-obstetrical patients	Moderate	Strong against
**Specific recommendations by some clinical specialties**		
**Renal recommendation**		
11. **Adjustments of insulin therapy in diabetic patients**		
50% reduction in long-acting insulin	Low	Strong for
Correction with short-acting insulin	Low	Strong for
Oral hypoglycemic agents	Low	Strong for
**Cardiovascular recommendations**		
12. **Beta-blockers**		
Continuation of beta-blockers	Low	Weak for
For patients with positive test for myocardial ischemia undergoing vascular surgery	Low	Weak for
Start the day of surgery treatment regardless of the condition to be treated	High	Strong against
13. **Statins**		
Continuation of statins or start before undergoing noncardiac surgery patients with significant atherosclerosis as secondary prevention	Low	Weak for
Treatment naïve patients undergoing noncardiac surgery without significant atherosclerosis	Low	Strong against
14. **Aspirin**		
Suspending aspirin three or more days before noncardiac surgery and not restart within a week after it	High	Strong for
Continuation of aspirin (75–100 mg daily) in patients who presented acute coronary syndrome in the last 12 months or history of percutaneous coronary intervention	Low	Weak for
Start or not to suspend treatment prior to surgery	High	Strong against
15. **Renin-angiotensin system inhibitors**		
Suspend them the day of surgery in chronically medicated patients and restart immediately in hemodynamically stable conditions	Low	Weak for
Start in patients with severe hypertension or ventricular dysfunction if suspending the day of surgery		
Start treatment the day of surgery in patients who do not receive it chronically	Low	Strong against
16. **Calcium channel blockers**		
Suspend the single preoperative dose the day of the surgery in chronically medicated patients	Low	Weak for
Starting treatment in patients with inducible myocardial ischemia or suspected coronary vasospasm during preoperative evaluation and suspend the single dose the day of surgery		
Starting calcium channel blockers in the preoperative surgery in patients who do not receive chronically	Low	Strong against

*MRSA* methicillin resistant *Staphylococcus aureus*

*The presented level of evidence and recommendation strength comes from the EB-CPG with the highest overall and methodological rigor AGREE-II score. The level of evidence and recommendation strength by EB-CPG are presented in the online supplemental material 8.a

We found discrepancies among EB-CPG in 3 out of 78 (4%) of the therapeutic/preventive care recommendations. After applying the direction and strength of recommendations rule to decide doing or not doing the CPG, 15 (55 specific) recommended and 10 (23 specific) did not recommended therapeutic/preventive interventions (see Online supplemental material Appendix 8, Online supplemental material Appendix 6 – Table 1 GRADE level of evidence and strength of recommendations by CPG; Table 2 Recommended therapeutic/preventive care only, and Table 3 Not recommended therapeutic/preventive care).

## Discussion

To the best of our knowledge, the present study is the first overview of guidelines encompassing a broad spectrum of preoperative care recommendations.

We observed higher level of evidence supporting therapeutic than risk stratification recommendations (high/moderate quality of evidence 59 vs 22%, respectively). It is not surprising because cross-sectional or cohort studies can provide high-quality evidence for test accuracy but indirect evidence for patient-important outcomes. Furthermore, highs level of heterogeneity is almost the rule in risk stratifications test, downgrading even more the level of evidence because of inconsistency [42–44].

The strength of a recommendation is defined as the extent to which one can be confident that the desirable effects of an intervention outweigh its undesirable ones. We found only 12/53 (23%) “strong” risk stratification recommendations statements (for and against) based on high/moderate level of evidence and 43/78 (55%) for therapeutic/preventive care recommendation. Although it would be desirable that higher proportions of high-quality supporting evidence guide panel must consider additional factors. In order to assess competing management alternatives, GRADE proposes to consider four domains: estimates of effect for desirable and undesirable outcomes, confidence in the estimates of effect, values and preferences, and resource use. Guideline panels must integrate these factors to make a strong or weak recommendation for or against an intervention [45].

After our search date, the updated guideline from the European Society of Anesthesiology (ESA) was published, using GRADE and searching until May 2016 [46]. This CPG addressed two main clinical questions in order to help each anesthesiologists in their daily practice: (1) how should a pre-operative consultation clinic be organized and (2) how should pre-operative assessment of a patient be performed. As in our present work, this guideline covered specific conditions that might adversely interfere with anesthesia and surgery, including cardiovascular disease, respiratory disease, smoking, obstructive sleep apnea syndrome, renal disease, diabetes, obesity, coagulation disorders, anemia and pre-operative blood conservation strategies, the geriatric patient, alcohol and drug misuse and addiction, and currently also neuromuscular disease. We are hereby presenting a preoperative clinical risk criteria and categories that were complemented with established risk factors for postoperative pulmonary complications (see Online supplemental material Appendix 3) [46]. The 2018 ESA guidelines also provided independent predictors for difficult mask ventilation, a topic not specifically addressed in previous CPGs [46].

As described, RCTs are still few and therefore many preoperative interventions rely to a large extent on expert opinion, which in turn requires to be adapted to the reality of nations’ healthcare systems. This large evidence gap should be addressed by related researchers in order to improve the certainty in evidence-based recommendations.

Studies on prognostic or diagnostic accuracy tests, including scoring of severity of illness, usually provide low quality of evidence, even when scores such as ASA-PS, RCRI, NSQIP-MICA, POSSUM, and others have been extensively validated [46].

Our updated overview of EB-CPGs, conducted under the rigorous Cochrane methods, may be a useful resource for the professionals involved in preoperative care to consult during decision-making. We present many strong recommendations with sufficient evidence to be routinely implemented in clinical practice. However, any decision should be taken considering local contextual factors.

In addition, cost reductions were identified at the clinical level as well as at the health system level in another study [10–12, 47]. Two guidelines also suggested strong costs benefits both for patients and society [48, 49]. Another study showed that the application of EB-CPGs significantly improved the efficiency of the preoperative evaluation without negatively affecting the quality of care [50]. These findings were consistent across different settings, like in a hospital in Barbados where the introduction of guidelines reduced the burden of presurgical tests and costs with not hampering patient’s safety [51]. In the same way, a recent study in a hospital in New Jersey, USA, found that approximately 25% of tests were not justifiable and could be thus eliminated by complying with NICE/ASA guidelines. The evaluation of applying these changes in practice showed significant savings without altering clinical outcomes [52].

Recommendations can be adopted, modified, or even not implemented, depending on institutional or national requirements and legislation and local availability of devices, drugs, and resources [53]. Decision-makers at the national and subnational levels should be provided with the information they need to apply the evidence and recommendations in their setting [54]. As a limitation, including only EB-CPGs could have resulted in omitting some information, but we prioritized summarizing the highest quality evidence. Our exclusion criteria for CPGs, limiting the scope, may represent an additional caveat. Our inclusion/exclusion criteria focused on general recommendations provided a lower amount of evidence for certain practices than if we had also included recommendations for single conditions, specific prophylaxis, or single body part surgeries. Such approach, however, would have compromised the feasibility of our systematic review due to the enormous number of such guidelines. Nonetheless, we provided detailed lists with numerous recommendations and reflected guideline’s discrepancies, suggesting that this could not have been a major limitation.

Our study will be useful for future preoperative care guideline developers or adapters. Consistently with other overviews of clinical guidelines, the domain that received the lowest mean score was the “applicability” domain of the AGREE-II tool. Similarly, the heterogeneity of evidence and the strength of recommendation grading systems in this overview echo that of other clinical guideline overviews [55–57]. Low scores in the applicability domain result in inadequate adoption rates of guidelines, particularly for preoperative care where “defensive medicine” (i.e., prescribing more tests than necessary just to prevent litigation) is very common. We also found some discrepancies, mainly in the evidence level, in each recommendation that did not always discriminate between universal interventions and those suitable only for special target groups or specific surgeries.

Guideline developers should ensure rigorous methodological processes and should also make recommendations that are formulated and disseminated in ways that facilitate understanding and application by end-users. For example, the DECIDE Collaboration conducted research and developed tools to improve implementation of evidence-based recommendations by different target audiences, including providers, policy makers, and the public [58]. In that sense, GRADE provides guideline developers with a comprehensive and transparent framework for grading quality of evidence and of strength of recommendations.

Our overview identified several controversies, evidence gaps, and issues regarding preoperative care guidelines that warrant future research and reveal opportunities to improve the guidelines quality.

For example, we found many discrepancies about risk stratification recommendations like electrocardiography and chest X-ray, polysomnography, assessment of left ventricular function, stress testing, and coronary angiography in certain populations. We found less discrepancies for therapeutic/preventive care mainly because antimicrobial prophylaxis use beta-blockers (find these discrepancies in the Online supplemental material Appendix 6 and Appendix 8**)**.

From the perspective of the anesthesiologist practice, there still remain many unanswered questions. For example, in the patient with significant medical, surgical, or obstetrical history, it would be useful to understand how early the pre-anesthetic evaluation should be performed, considering the time required to optimize the patient’s status. There are also uncertainties for the recommendation of fasting for solids in adults and children since many factors can delay gastric emptying, and no fixed rules apply. Fasting should be individualized in some patients and depend on the characteristics of the fat intake. Regarding prokinetics and antacids, patients’ comorbidities like esophageal pathology, bariatric surgery history, or obesity should be considered in the decision, but there is no formal recommendation. In the same way, suspending or not suspending aspirin should be evaluated according to the patient’s history and risk of bleeding of the surgery that could be catastrophic in neurosurgery, spinal surgery, or ophthalmologic surgery. It is also strange that informed consent only has a “weak for,” recommendation from a unique CPGs since there is enough background of litigation due to the lack of consent.

We encourage guideline developers to adopt GRADE and AGREE-II tools to elaborate future sound preoperative care guidelines [7, 22].

The huge amount of resources involving preoperative care warrants high-quality nationwide EB-CPGs supported by all relevant stakeholders to improve the chances of a successful implementation. This probably includes the involvement of the Ministry of Health, scientific societies, and consumers working together through a formal process of implementation and monitoring [17, 59].

Although standardization of preoperative care may be desirable, differences in recommendations could reflect differences in contextual factors such as organizational or financial arrangements, legal framework, varied values and preferences, and the acceptability and feasibility of using different interventions. Research exploring reasons for conflicting recommendations in different countries or settings could also drive overall improvements in guideline quality. The key findings are described in Table 4.

**Table 4:** Key points

• The included evidence-based clinical practice guidelines (EB-CPGs) showed significant heterogeneity both of evidence and recommendation grading systems; GRADE was the most commonly used. • About half of the included EB-CPGs were updated in the last 5 years, and one third of them were rated as strongly recommended based in their high AGREE-II performance. • They were generally deficient in applicability and in providing monitoring tools. • We summarized 31 risk stratification and 16 therapeutic/preventive recommendations. • We found 93 strong for and 46 strong against recommendations, all of which were ready to be considered to be implemented or to be interrupted, respectively. • The level of evidence and strength of recommendation was higher for therapeutic/preventive recommendation than for risk stratification ones. • We only found 12/53 (55%) strong risk stratification recommendations based on high/moderate level of evidence and 43/78 (55%) for therapeutic/preventive care recommendations.	

In conclusion we found significant heterogeneity of guidelines’ quality and rating systems, as well as deficiencies in several guideline quality domains, which reveal opportunities for quality improvement which deserve careful consideration by future guideline developers. Nevertheless, we present many strong recommendations ready to be at present considered for implementation or discontinuation.

## Supplementary information

**Additional file 1.** Online Only Supplemental Material.

## Data Availability

Not applicable.

## Abbreviations

CPG: Clinical practice guidelines
GRADE: Grading of Recommendations Assessment, Development, and Evaluation
HTA: Health Technology Assessment
EB-CPG: Evidence based-clinical practice guidelines
RCT: Randomized clinical trials
Y: Yes
N: No
PY: Probably yes
PN: Probably no
HSTAT: Health services/technology assessment text
NICE: National Institute for Health and Clinical Excellence
ICSI: Institute for Clinical Systems Improvement
ESA: European Society of Anesthesiology
ESC/ESA: European Society of Cardiology/European Society of Anesthesia
SBC: Brazilian Society of Cardiology
ACC/AHA: American College of Cardiology/American Heart Association
CCSG: Canadian Cardiovascular Society
SARNePI: Italian Society of Anesthesia and Intensive Pediatric Therapy and Neonatology
ICSI: Institute for the Improvement of Clinical Systems
BARA: Regional Anesthesia Associations of Belgium
ASHP: American Society of Health-System Pharmacists
SIGN: Scottish Intercollegiate Guidelines Network
SAC: Argentine Society of Cardiology
EROS: Early Review Organizing Software

## Footnotes

1 https://www.iecs.org.ar/wp-content/uploads/Protocolo-de-Overview-y-adaptaci%C3%B3n-de-GPC-prequir%C3%BArgicas-1.pdf
