# Supplementary material for: The quality of clinical practice guidelines for preoperative care using the AGREE II instrument: a systematic review
Source: Syst Rev. 2020 Jul 13;9:159. doi: 10.1186/s13643-020-01404-8 (PMC7359265; doi:10.1186/s13643-020-01404-8)
Supplement: Supplementary file 1 — Additional file 1. Online Only Supplemental Material. [file 13643_2020_1404_MOESM1_ESM.docx]

**ONLINE ONLY SUPPLEMENTAL MATERIAL**

**Overview of evidence-based clinical practice guidelines for preoperative care: a systematic review**

Ciapponi A, Tapia López E, Virgilio SA, Bardach A.

Institute for Clinical Effectiveness and Health Policy (IECS-CONICET), Buenos Aires, Argentina.

**Appendix 1**

**Table 1. e-PRISMA checklist 1 Overview of evidence-based clinical practice guidelines for preoperative care: a systematic review**

| **Section/topic** | **#** | **Checklist item** | **Reported on page #** |
| --- | --- | --- | --- |
| **TITLE** | | |  |
| Title | 1 | Identify the report as a systematic review, meta-analysis, or both. | 1 |
| **ABSTRACT** | | |  |
| Structured summary | 2 | Provide a structured summary including, as applicable: background; objectives; data sources; study eligibility criteria, participants, and interventions; study appraisal and synthesis methods; results; limitations; conclusions and implications of key findings; systematic review registration number. | 1 |
| **INTRODUCTION** | | |  |
| Rationale | 3 | Describe the rationale for the review in the context of what is already known. | 3 |
| Objectives | 4 | Provide an explicit statement of questions being addressed with reference to participants, interventions, comparisons, outcomes, and study design (PICOS). | 3 |
| **METHODS** | | |  |
| Protocol and registration | 5 | Indicate if a review protocol exists, if and where it can be accessed (e.g., Web address), and, if available, provide registration information including registration number. | 3 |
| Eligibility criteria | 6 | Specify study characteristics (e.g., PICOS, length of follow-up) and report characteristics (e.g., years considered, language, publication status) used as criteria for eligibility, giving rationale. | 3 |
| Information sources | 7 | Describe all information sources (e.g., databases with dates of coverage, contact with study authors to identify additional studies) in the search and date last searched. | 3-4 |
| Search | 8 | Present full electronic search strategy for at least one database, including any limits used, such that it could be repeated. | 3-4 |
| Study selection | 9 | State the process for selecting studies (i.e., screening, eligibility, included in systematic review, and, if applicable, included in the meta-analysis). | 4 |
| Data collection process | 10 | Describe method of data extraction from reports (e.g., piloted forms, independently, in duplicate) and any processes for obtaining and confirming data from investigators. | 4 |
| Data items | 11 | List and define all variables for which data were sought (e.g., PICOS, funding sources) and any assumptions and simplifications made. | 4-5 |
| Risk of bias in individual studies | 12 | Describe methods used for assessing risk of bias of individual studies (including specification of whether this was done at the study or outcome level), and how this information is to be used in any data synthesis. | 4-5 |
| Summary measures | 13 | State the principal summary measures (e.g., risk ratio, difference in means). | 6 |
| Synthesis of results | 14 | Describe the methods of handling data and combining results of studies, if done, including measures of consistency (e.g., I^2^) for each meta-analysis. | NA |
| Risk of bias across studies | 15 | Specify any assessment of risk of bias that may affect the cumulative evidence (e.g., publication bias, selective reporting within studies). | 4-5 |
| Additional analyses | 16 | Describe methods of additional analyses (e.g., sensitivity or subgroup analyses, meta-regression), if done, indicating which were pre-specified. | NA |
| **RESULTS** | | |  |
| Study selection | 17 | Give numbers of studies screened, assessed for eligibility, and included in the review, with reasons for exclusions at each stage, ideally with a flow diagram. | 5, 13 |
| Study characteristics | 18 | For each study, present characteristics for which data were extracted (e.g., study size, PICOS, follow-up period) and provide the citations. | Table 1 |
| Risk of bias within studies | 19 | Present data on risk of bias of each study and, if available, any outcome level assessment (see item 12). | Page 5-6 |
| Results of individual studies | 20 | For all outcomes considered (benefits or harms), present, for each study: (a) simple summary data for each intervention group (b) effect estimates and confidence intervals, ideally with a forest plot. | Table 1, |
| Synthesis of results | 21 | Present results of each meta-analysis done, including confidence intervals and measures of consistency. | Suppl. |
| Risk of bias across studies | 22 | Present results of any assessment of risk of bias across studies (see Item 15). | Suppl. |
| Additional analysis | 23 | Give results of additional analyses, if done (e.g., sensitivity or subgroup analyses, meta-regression [see Item 16]). | NA |
| **DISCUSSION** | | |  |
| Summary of evidence | 24 | Summarize the main findings including the strength of evidence for each main outcome; consider their relevance to key groups (e.g., healthcare providers, users, and policy makers). | 6-7 Box 1 |
| Limitations | 25 | Discuss limitations at study and outcome level (e.g., risk of bias), and at review-level (e.g., incomplete retrieval of identified research, reporting bias). | 7-8 |
| Conclusions | 26 | Provide a general interpretation of the results in the context of other evidence, and implications for future research. | 9 |
| **FUNDING** | | |  |
| Funding | 27 | Describe sources of funding for the systematic review and other support (e.g., supply of data); role of funders for the systematic review. | 10 |

*From:*  Moher D, Liberati A, Tetzlaff J, Altman DG, The PRISMA Group (2009). Preferred Reporting Items for Systematic Reviews and Meta-Analyses: The PRISMA Statement. PLoS Med 6(6): e1000097. doi:10.1371/journal.pmed1000097.

**Appendix 2**

**Table 1 - Search strategy**

| **Medline** | |
| --- | --- |
| 1. | surgical procedures, elective/ |
| 2. | elective surg*.ti. |
| 3. | ambulatory surgical procedures/ |
| 4. | ambulatory surg*.ti. |
| 5. | exp ambulatory care/ |
| 6. | preoperative care/ |
| 7. | preoperative period/ |
| 8. | perioperative nursing/ or perioperative care/ or perioperative period/ |
| 9. | (pre-operative* or preoperative* or preop or pre-op or pre-surg* or presurg* or perioperative* or peri-operative*).ti. |
| 10. | ((before or prior or advance) adj4 (surg* or operat* or anaesthes* or anesthes* or elective or ambulatory)).ti. |
| 11. | (day adj2 surg*).ti. |
| 12. | outpatient surg*.ti. |
| 13. | or/1-12 |
| **Embase** | |
| 1. | *elective surgery/ |
| 2. | elective surg*.ti. |
| 3. | *ambulatory surgery/ |
| 4. | ambulatory surg*.ti. |
| 5. | *ambulatory care/ |
| 6. | *preoperative care/ or *preoperative evaluation/ |
| 7. | *preoperative period/ |
| 8. | *perioperative period/ |
| 9. | (pre-operative* or preoperative* or preop or pre-op or pre-surg* or presurg* or perioperative* or peri-operative*).ti. |
| 10. | ((before or prior or advance) adj4 (surg* or operat* or anaesthes* or anesthes* or elective or ambulatory)).ti. |
| 11. | (day adj2 surg*).ti. |
| 12. | outpatient surg*.ti. |
| 13. | or/1-12 |
| **Cochrane** | |
| #1. | mesh descriptor: [surgical procedures, elective] this term only |
| #2. | elective surg*:ti |
| #3. | mesh descriptor: [ambulatory surgical procedures] this term only |
| #4. | ambulatory surg*:ti |
| #5. | mesh descriptor: [ambulatory care] explode all trees |
| #6. | mesh descriptor: [preoperative care] this term only |
| #7. | mesh descriptor: [preoperative period] this term only |
| #8. | mesh descriptor: [perioperative nursing] this term only |
| #9. | mesh descriptor: [perioperative care] this term only |
| #10. | mesh descriptor: [perioperative period] this term only |
| #11. | (pre-operative* or preoperative* or preop or pre-op or pre-surg* or presurg* or perioperative* or peri-operative*):ti |
| #12. | ((before or prior or advance) near/4 (surg* or operat* or anaesthes* or anesthes* or operat* or elective or ambulatory)):ti |
| #13. | (day near/2 surg*):ti |
| #14. | outpatient next surg*:ti |
| #15. | ^2-#14^ |
| **LILACs** | |
| MH Procedimientos Quirrdimien Electivos OR ((Electiv$ OR Opcion$ OR Optativ$ OR Eletiv$) AND (Ciru$ OR Surger$)) OR MH Procedimientos Quirrdimien Ambulatorios OR ((Cir ($ OR Quirurg$ OR Surgical OR Surger$ OR Cirugia$) AND (Ambulat(A$)) OR MH Atenci M Ambulatoria OR ((AtenciAt OR Cuidado$ OR Assistssist OR Care$) AND (Ambulator$)) OR MH Periodo Perioperatorio OR MH Cuidados Preoperatorios OR MH Enfermernf Perioperatoria OR Preopera$ OR PrOR Pra$ OR$ OR Prequirur$ OR Pre-Quirur$ OR Perioperat$ OR Pre-Surg$ OR Presurg$ OR Peri-Operat$ OR ((Pre OR before OR prior OR advance OR Previa OR Posterior$) AND (surg$ OR operat$ OR anaest$ OR antes$ OR Electiv$ OR ambulator$)) | |

**Filter for guidelines:** ((Guideline*[ti] OR Search Practice Guideline[pt] OR Search Guideline[pt])

**Appendix 3**

**Table 1 a. Preoperative clinical risk criteria and categories**

| **Major criteria:**   1. Urgent or emergency surgery 2. Recent high-risk coronary heart disease (acute myocardial infarction, acute coronary syndrome or angina in functional class 3 or 4 in the 6 months prior to surgery) 3. Severe aortic or mitral stenosis |
| --- |
| **Minor criteria:**   1. Age > 70 years 2. Diabetes Mellitus 3. History of peripheral vascular disease 4. History of stable coronary disease 5. History of congestive heart failure or ejection fraction <40% 6. History of stroke 7. Severe mitral or aortic regurgitation 8. Severe COPD 9. Active cancer 10. Chronic renal failure (creatinine ≥ 2.0 mg/dL) |
| **Preoperative clinical risk categories**   1. **High clinical risk**    1. If there are one or more major criteria, regardless of the existence of minor criteria    2. If there are two minor criteria 2. **Moderate clinical risk:** If there is one minor criterion or none greater 3. **Low clinical risk:** There is no major or minor criterion |

**Table 1.b Risk factors** **for postoperative pulmonary complications**

| **Risk factor** | **OR** | **95% CI** |
| --- | --- | --- |
| Age 60 to 69 years^a^ | 2.09 | 1.70 to 2.48 |
| Age 70 to 79 years^a^ | 3.04 | 2.11 to 4.39 |
| COPD | 1.79 | 1.44 to 2.22 |
| Smoking | 1.26 | 1.01 to 1.56 |
| Congestive heart failure | 2.93 | 1.02 to 8.43 |
| Total functional dependence | 2.51 | 1.99 to 3.15 |
| Partial functional dependence | 1.64 | 1.36 to 2.01 |
| Higher ASA classification and prolonged duration of surgery | 2.14 | 1.33 to 2.46 |

**ASA**, American Society of Anesthesiology; **CI**, confidence interval; **COPD**, chronic obstructive pulmonary disease; OR, odds ratio. a Compared with patients aged <60 years.

**Source** De Hert, S., Staender, S., Fritsch, G., Hinkelbein, J., Afshari, A., Bettelli, G.Wappler, F. (2018). Pre-operative evaluation of adults undergoing elective noncardiac surgery: Updated guideline from the European Society of Anaesthesiology. European Journal of Anaesthesiology (EJA), 35(6), 407-465. doi:10.1097/eja.0000000000000817

**Appendix 4.**

**Table 1. Guidelines’ Evidence and Recommendation Grading Systems**

| **Guide / System** | **Level of the Evidence** | **Strength of the Recommendation** |
| --- | --- | --- |
| -NICE 2016[^33^](#_ENREF_33)  -CDC 2017[^46^](#_ENREF_46)  -ERAS Society 2016[^41^](#_ENREF_41)  -ERAS Society 2012[^38^](#_ENREF_38)  -CCSG 2017[^37^](#_ENREF_37)  -ICSI 2012[^40^](#_ENREF_40)  **-**SAC 2016[^48^](#_ENREF_48) (QoE)  **GRADE** | **High ⊕⊕⊕⊕**: Further research is very unlikely to change our confidence in the estimate of effect.  **Moderate ⊕⊕⊕🌕**: Further research is likely to have an important impact on our confidence in the estimate of effect and may change the estimate.  **Low ⊕⊕🌕🌕**: Further research is very likely to have an important impact on our confidence in the estimate of effect and is likely to change the estimate.  **Very low ⊕🌕🌕🌕**: We are very uncertain about the estimate. | **Strong** **for** **↑↑**:   the panel considers that the desirable effects of adhering to a recommendation clearly outweigh the undesirable effects  **Strong** **against** **↓↓**:   vice versa.  **Weak** **for** **↑**: The panel considers that the desirable effects of adhering to a recommendation exceed the undesirable effects, although there is uncertainty.  **Weak** **against** **↓**:   vice versa. |
| -ESC/ESA 2014[^34^](#_ENREF_34)  **-**SAC 2016[^48^](#_ENREF_48) (for recommendation strength)  **Own system** | **A**: Data from multiple RCTs in the meta-analysis.  **B**: Data from a single RCT or from large non-randomized studies.  **C**: Consensus of opinion of experts and/or small studies, retrospective studies, registries. | **Class I**: Evidence and/or general agreement that a certain diagnostic/treatment procedure It is beneficial, useful and effective.  **Class II**: Conflicting evidence and/or divergence of opinion about the usefulness/effectiveness of the treatment.  **-Class IIa**: The weight of the evidence/opinion is in favor of utility/effectiveness.  **-Class IIb**: Utility/effectiveness is less established by evidence/opinion.  **Class III**: Evidence or general agreement that the treatment is not useful/effective and in some cases, may be harmful |
| -ACC/AHA 2014[^36^](#_ENREF_36)  -SBC 2017[^35^](#_ENREF_35)  **Own system** | **A**: Evidence coming from of RCTs and meta-analysis from several populations.  **B**: Evidence from a limited group of populations and from a single RCT or of non-randomized clinical trials.  **C**: Evidence from of a very limited number of populations and consensus or expert opinions, reports and case series group. | **Class I**. **Benefit Risk >>>** **(should be administered):** Evidence and/or general agreement that n treatment or procedure is beneficial, useful and effective.  **-** **Class IIa**. **Benefit >> Risk** **(REASONABLE to administer)**: Contradictory evidence and/or divergent opinion on the benefit of the procedure, but the evidence supports that the treatment/procedure can help the patient.  - **Class IIb**. **Benefit ≥ Risk** **(MAY BE CONSIDERED):** Contradictory evidence and/or diverging opinion on the benefit of the procedure and is not well defined if the treatment/procedure can help the patient.  **Class III**. **Risk ≥ Benefit** **(SHOULD NOT be administered)**: Evidence or general agreement that the treatment or procedure given It is not useful/effective, and in some cases, it can be harmful. |
| -SARNePi 2014[^39^](#_ENREF_39)  -PNLG 2009[^47^](#_ENREF_47)  **Own system** | **I**: Evidence obtained from multiple RCTs and/or systematic reviews of RCTs.  **II**: Evidence from a single RCT with proper design  **III**: Evidence obtained from cohort studies with simultaneous or historical controls or their meta-analysis.  **IV**: Evidence of retrospective case-control studies or their meta-analysis.  **V**: Evidence obtained from case series studies without control groups  **VI**: Evidence based on the opinion of experts, or based on opinions of the members of the working group responsible for the guide | **A**: It is strongly recommended to perform a diagnostic procedure or test in particular. supported by scientific evidence of good quality, although not necessarily type I or II.  **B**: Uncertainty that the procedure or intervention should always be recommended, and the execution must be carefully considered.  **C:** There is great uncertainty for or against regarding the recommendation to perform the procedure or intervention.  **D**: The execution of the procedure is not recommended.  **E**: The procedure is strongly discouraged. |
| -SIGN 2014[^45^](#_ENREF_45)  -ESA 2011[^42^](#_ENREF_42)  **SIGN** | **1++**: High quality meta-analysis, systematic reviews of RCTs or RCTs with very low risk of bias  **1+**: Well conducted meta-analyzes, systematic reviews or RCTs with low risk of bias  **1-**: Meta-analyzes, systematic reviews or RCTs with a high risk of bias  **2++**: High-quality systematic reviews of case-control or cohort studies  **2+**: Well- conducted cohort or case-control studies, with low risk of confusion or bias and a moderate  **2-**: Case-control studies or cohort with high risk of confusion or bias and a significant risk that relationship is not causal  **3**: Non-analytical studies such as case reports or case series  **4**: Opinion of experts. | **A**: At least one meta-analysis, systematic review or RCT rated as 1 ++ directly applicable to the target population; or a body of evidence consists mainly of 1+ studies, directly applicable to the target population and demonstrating the general consistency of the results.  **B**: A body of evidence that includes 2++ studies, directly applicable to the target population and demonstrating overall consistency of results; or  extrapolated evidence from 1++ or 1+ studies.  **C**: A body of evidence that includes 2+ studies, directly applicable to the target population and demonstrating overall consistency of results; or extrapolated evidence from 2++ studies.  **D**: Level of evidence 3 or 4; or extrapolated evidence from 2+ studies. |
| -BARA 2013[^43^](#_ENREF_43)  **Own system** | **A**: High quality of evidence. Evidence obtained from at least one correctly performed meta-analysis or from an adequately conducted RCT.  **B**: Moderate quality of evidence. Evidence obtained from non-randomized clinical trials or other types of quasi – experimental studies.  **C**: Low quality of evidence. Evidence obtained from descriptive studies such as comparative studies, correlation studies, case - control studies and retrospective studies.  **D**: Very low quality of evidence. Opinions of respected authorities in the field or reports of expert committees. | **1. Strong**: The desirable effects of adhering to a recommendation clearly outweigh the undesirable effects  **2.** **Weak**: the desirable effects of adhering to a recommendation probably outweigh the undesirable, although there is uncertainty. |
| -ASHP 2013[^44^](#_ENREF_44)  **Own system** | **I**: Evidence obtained from RCTs large well-conducted or meta-analysis.  **II**: Evidence obtained from RCTs small well-led  **III**: Evidence obtained from well-conducted cohort studies.  **IV**: Evidence of well - conducted case - control studies.  **V**: Evidence obtained from well-conducted uncontrolled studies.  **VI**: Conflicting evidence that tends to favor the recommendation.  **V** **I** **I**: Expert opinion or extrapolated data. | **A**: Based on levels of visibility I-III.  **B**: Based on levels of vision IV-VI.  **C**: Based on level of vision VII. |

**Appendix 5**

**Table 1. AGREE-II domain scores expressed as a percentage**

| **Guide - year of publication** | **AGREE-II domain scores** (From 0 to 100%) | | | | | | **Guideline Quality**  1=lowest 7= highest | **Recommended**  (++, +, -) |
| --- | --- | --- | --- | --- | --- | --- | --- | --- |
|  | Scope & Purpose | Stakeholders involvement | Rigor in the elaboration | Clarity of presentation | Applicability | Editorial independence |  |  |
| NICE 2016[^33^](#_ENREF_33) | 86 | 58 | 82 | 97 | 56 | 91 | **6** | **++** |
| ESC/ESA 2014[^34^](#_ENREF_34) | 91 | 63 | 41 | 86 | 31 | 83 | **4.5** | **+** |
| SBC 2017[^35^](#_ENREF_35) | 88 | 50 | 30 | 94 | 14 | 91 | **4.5** | **+** |
| ACC/AHA 2014[^36^](#_ENREF_36) | 91 | 52 | 65 | 97 | 20 | 100 | **6** | **+** |
| CCSG 2017[^37^](#_ENREF_37) | 41 | 47 | 30 | 70 | 12 | 96 | **3** | **+** |
| ERAS Society 2012[^38^](#_ENREF_38) | 38 | 36 | 45 | 69 | 6 | 100 | **4** | **+** |
| SARNePi 2014[^39^](#_ENREF_39) | 83 | 47 | 30 | 69 | 6 | 75 | **4** | **+** |
| ICSI 2012[^40^](#_ENREF_40) | 77 | 61 | 35 | 66 | 18 | 100 | **4** | **+** |
| ERAS Society 2016[^41^](#_ENREF_41) | 61 | 41 | 52 | 86 | 12 | 79 | **4** | **+** |
| ESA 2011[^42^](#_ENREF_42) | 86 | 58 | 72 | 62 | 12 | 87 | **4** | **++** |
| BARA 2013[^43^](#_ENREF_43) | 69 | 33 | 35 | 66 | 0 | 37.5 | **3** | **+** |
| ASHP 2013[^44^](#_ENREF_44) | 75 | 72 | 79 | 100 | 58 | 100 | **5.5** | **++** |
| SIGN 2014[^45^](#_ENREF_45) | 89 | 83 | 97 | 100 | 87 | 100 | **6.5** | **++** |
| CDC 2017[^46^](#_ENREF_46) | 97 | 72 | 90 | 97 | 31 | 100 | **6** | **++** |
| PNLG 2009[^47^](#_ENREF_47) | 72 | 39 | 39 | 83 | 6 | 17 | **3** | **+** |
| SAC 2016[^48^](#_ENREF_48) | 58 | 53 | 56 | 82 | 23 | 82 | **6** | **++** |
| **Median** (P 25-75**)** | 80 (65-89) | 53 (44-62) | 49 (35-76) | 85 (69-97) | 16 (9-31) | 91 (81-100) | 4 (4-6) |  |
| **Mean** (SD) | **75** (18) | **54** (14) | **55** (23) | **83** (14) | **25** (24) | **84** (24) | **5** (1) |  |
| **Minimum-Maximum** | 38-97 | 33-83 | 30-97 | 62-100 | 0-87 | 17-100 | 3-7 |  |

++: Strongly recommended, +: Recommended, -: Not recommended; SD: Standard Deviation; P 25-75: Percentile 25-75%.

Scope & Purpose domain considered the following items: a) The general objective (s) of the guide is specifically described; b) The health aspect (s) covered by the guide is specifically described; c) The population (patients, public, etc.) to which the guide is intended to apply is specifically described.

Stakeholders involvement domain considered the following items: a) The group that develops the guide includes individuals from all relevant professional groups; b) The views and preferences of the target population (patients, public, etc.) have been taken into account; c) The target users of the guide are clearly defined.

Rigor in the elaboration domain considered the following items: a) Systematic methods have been used to search for evidence; b) The criteria for selecting the evidence are clearly described; c) The strengths and limitations of the evidence set are clearly described; d) The methods used to formulate the recommendations are clearly described; e) In formulating the recommendations, health benefits, side effects and risks have been considered; f) There is an explicit relationship between each of the recommendations and the evidence on which they are based; g) The guide has been reviewed by external experts before its publication; h)  A procedure is included to update the guide.

Clarity of presentation domain considered the following items: a) The recommendations are specific and are not ambiguous; b) The different options for the management of the disease or health condition are clearly presented; c) The key recommendations are easily identifiable.

Applicability domain considered the following items: a) The guide describes facilitators and barriers for application; b) The guide provides advice and / or tools on how the recommendations can be put into practice; c) The possible implications of the application of recommendations on resources have been considered; d) The guide provides criteria for monitoring and / or audit of the recommendations.

Editorial independence domain considered the following items: a) The views of the funding entity have not influenced the content of the guide; b) Conflicts of interests of the members of the group that developed the guide have been registered and addressed.

**Appendix 6**

**Table 1.** **Risk stratification evaluations, GRADE level of evidence and strength of recommendations by CPG: Do?**

| **Recommendation** | **Guide** | **Level of Evidence** | **Strength of Recommendation** | **Do*** |
| --- | --- | --- | --- | --- |
| 1. **Electrocardiography:** | | | |  |
| In neonates and/or children of 6 months | SARNEPI 2014 | Low | Weak for |  |
| Healthy people undergoing minor surgery | NICE 2016:  SBC 2017; SARNEPI 2014 | Low | Strong against | N |
|  | ESC/ESA 2014 | Moderate |  |  |
|  | ICSI 2012 | High |  |  |
|  | SAC 2016 | Moderate | Weak against |  |
| Perform in cases of clinical suspicion | SARNEPI 2014 | Low | Weak for |  |
| People over 65 undergoing minor or intermediate surgery | ICSI 2012;  SBC 2017 | Low | Weak for | ? |
|  | NICE 2016 | Very low | Strong against |  |
|  | ESC/ESA 2014 | Very low | Weak against |  |
| People with cardiovascular disease | ACC/AHA 2014 | Moderate | Weak for | PY |
|  | SBC 2017 | Low | Strong for |  |
|  | NICE 2016;  SAC 2016 | Low | Weak for |  |
| People with a morbidity undergoing intermediate or major surgery | ESC/ESA 2014 | Very low | Strong for | Y |
|  | SAC 2016 | High |  |  |
|  | SBC 2017 | Low |  |  |
| 1. **Effort electrocardiography** | | | |  |
| Patients undergoing surgeries of intermediate or high risk of complications, including arterial vascular surgery (without severe cardiovascular perioperative conditions) | SBC 2017 | Low | Weak for |  |
| Patients undergoing low-risk surgery | SBC 2017 | Low | Strong against |  |
| Patients undergoing intermediate-risk surgery | SBC 2017 | Low | Strong against |  |
| 1. **Resting echocardiography** | | | |  |
| **High-risk surgery** | | | |  |
| Patient with suspected moderate or severe valvular involvement without evaluation in the last year or with worsening of symptoms | SBC 2017 | Low | Strong for |  |
| Patient with heart failure or symptoms suggestive of heart problems, without assessment in the past year, undergoing cardiac surgery | SBC 2017 | High | Strong for |  |
|  | SAC 2016 | Low | Weak for |  |
| Symptomatic patients with stent grafts who go to surgery and who have no evaluation in the last year | SBC 2017 | Low | Weak for |  |
|  | SAC 2016 | Low | Strong for |  |
| Asymptomatic patients | SBC 2017 | Low | Weak for |  |
| **Low, intermediate or uncertain surgical risk** | | | |  |
| Routine test in asymptomatic patients without suspect of heart failure or severe valvular disease | SBC 2017 | Low | Weak against | PN |
|  | NICE 2016; CCSG 2017 | Very low |  |  |
|  | SAC 2016 | Low | Strong against |  |
| 1. **Effort echocardiography** | | | |  |
| Routinely to assess cardiac risk | CCSG 2017 | Low | Strong against |  |
| 1. **Complete laboratory** | | | |  |
| Patients undergoing low-risk surgery independently of their ASA score | NICE 2016 | Very low | Strong against |  |
| Patients undergoing intermediate-risk surgery | NICE 2016 | Very low | Strong against |  |
| Patients with renal or cardiovascular disease undergoing intermediate-risk surgery that has not been recently evaluated | NICE 2016 | Very low | Weak for |  |
| Patients undergoing high-risk surgery | NICE 2016 | Very low | Strong for |  |
| Patients with preeclampsia or other preceding or a suspect of hemostatic disorder, it is recommended to apply platelet count, liver function test and evaluation of coagulation | BARA 2013 | Very low | Weak for |  |
| In case of bleeding or complications history of previous alloimmunization, it is recommended to evaluate the blood type. | BARA 2013 | Very low | Weak for |  |
| 1. **Tomographic coronary angiography** | | | |  |
| Routinely to assess cardiac risk | CCSG 2017 | Moderate | Strong against |  |
| 1. **Urinalysis** | | | |  |
| Routinely before surgery | NICE 2016 | Very low | Weak against |  |
| Urine or culture if diagnosing an urinary infection can influence surgery decisions | NICE 2016 | Very low | Weak for |  |
| 1. **Hemostasis/Coagulation Tests** | | | |  |
| Pediatric patients with negative history | SARNEPI 2014 | Low | Strong against |  |
| Patients with a history of bleeding | SBC 2017 | Low | Strong for |  |
| Patients with liver failure | SBC 2017 | Low | Strong for |  |
|  | NICE 2016 | Very low |  |  |
| In anticoagulated patients (e.g., consume Warfarin) | SBC 2017 | Low | Strong for |  |
|  | NICE 2016 | Very low |  |  |
| Patients with potential risk of bleeding undergoing intermediate or high-risk surgery | SBC 2017  ICSI 2012 | Low | Strong for | Y |
|  | NICE 2016 | Very low |  |  |
|  | SARNEPI 2014 | Low | Weak for |  |
| Routinely | NICE 2016 | Very low | Strong against |  |
| 1. **Hematocrit and hemoglobin** | | | |  |
| In pediatric patients with possible bleeding | SARNEPI 2014 | Low | Strong for |  |
| In pediatric patients routinely perform minor surgery | SARNEPI 2014 | Low | Strong against |  |
| Patients with anemia or blood disease or liver disease; when you suspected of anemia or other chronic disease during clinical examination. In medium or high-risk surgeries anticipated transfusion requirement | SBC 2017;  ICSI 2012 | Low | Strong for |  |
| Patients requiring intermediate or major surgery, and bleeding risk of transfusion requirement | SBC 2017 | Low | Strong for |  |
| Patients over 40 years | SBC 2017 | Low | Weak for |  |
| Patient s with a history of hematological or liver disease | SBC 2017 | Low | Strong for |  |
| 1. **Glycated hemoglobin (HbA1c) test** | | | |  |
| Diabetic patient without Hb1Ac within 3 months | NICE 2016 | Very low | Weak for |  |
| Patients without diabetes | NICE 2016 | Very low | Weak against |  |
| 1. **Polysomnography** | | | |  |
| In patients requiring continuous positive airway pressure (CPAP) | ICSI 2012 | High | Strong for |  |
| Patients presumed to have obstructive sleep apnea (OSA) based on the preoperative history and physical examination | ICSI 2012 | Low | Weak for | ? |
|  | NICE 2016 | Insufficient | - |  |
| 1. **Preoperative evaluation** | | | |  |
| Pediatric patients receiving anesthesia | SARNEPI 2014 | Very low | Strong for |  |
| Emergency surgeries in pediatric patients | SARNEPI 2014 | Very low | Strong against |  |
| All patients who undergoing diagnostic or therapeutic procedures | ICSI 2012 | Very low | Weak for |  |
|  | BARA 2013 | Low |  |  |
| Patients with ASA 1 or 2 without surgical or obstetric history (preanesthetic evaluation, including physical examination, the day of the procedure). | BARA 2013 | Very low | Weak for |  |
| Patient with significant medical, surgical or obstetrical history (anesthesiologist assessment) | BARA 2013 | Very low | Weak for |  |
| 1. **Assessment of left ventricular function** | | | |  |
| Patients suspected to have valvular disease with important clinical manifestations or undergoing liver transplantation | SBC 2017 | Moderate | Strong for | PY |
|  | ACC/AHA 2014 | Low | Weak for |  |
| Patients with heart failure without ventricular function assessment | SBC 2017 | Low | Weak for | ? |
|  | ACC/AHA 2014 |  | Weak against |  |
| Patients undergoing high-risk surgery | SBC 2017 | Moderate | Weak for |  |
| Obese patients (BMI ≥40) undergoing bariatric surgery | SBC 2017 | Low | Weak for |  |
| Routinely | ACC/AHA 2014 | Moderate | Strong against |  |
| 1. **Assessment of risk factors for surgical site infection** | | | |  |
| Assessment of smoking, diabetes, obesity, malnutrition and chronic skin disease | ICSI 2012 | Low | Strong for |  |
| 1. **Glucose** | | | |  |
| Routinely to pediatric patients | SARNEPI 2014 | Low | Strong against |  |
| Diabetic patients | ICSI 2012 | Low | Strong for |  |
| 1. **Informed consent** (Ideally written) | | | |  |
| Provide information on risks and benefits related to obstetric anesthesia and analgesia. | BARA 2013 | Very low | Weak for |  |
| 1. **Natriuretic peptide** | | | |  |
| Patients undergoing cardiac surgery | SBC 2017 | High | Weak for |  |
| Patients over 55 years with at least one cardiovascular risk factor undergoing non-cardiac surgery | SBC 2017 | Low | Weak for |  |
| 1. **Brain natriuretic peptide (BNP) or NT-proBNP** | | | |  |
| Patients over 65 years or patients between 45 and 64 years with significant cardiovascular disease or score (revised cardiac risk index (RCRI) ≥ 1 | CCSG 2017 | Moderate | Strong for |  |
| 1. **Lung function tests** | | | |  |
| Spirometry in patients undergoing non-high-risk surgery | NICE 2016 | Very low | Strong against |  |
| Arterial blood gas analysis in patients undergoing non-high-risk surgery | NICE 2016 | Very low | Strong against |  |
| Assessment by medical senior anesthesiologist after confirming respiratory illness or suspected in patients ASA 3/4 undergoing high-risk surgery | NICE 2016 | Very low | Weak for |  |
| 1. **Kidney function tests** | | | |  |
| For minor surgery in ASA 1/2 patients or intermediate-risk surgery in ASA 2 patients | NICE 2016 | Very low | Weak against |  |
| For complex or major surgery in ASA 1 patients at risk of acute kidney injury (AKI) | NICE 2016 | Very low | Weak for |  |
| In intermediate-risk surgery in ASA 2 patients at risk of AKI. In patients with increased risk surgery performed | NICE 2016 | Very low | Weak for |  |
| ASA 3/4 patients: at risk of AKI in low-risk surgery or just higher-risk surgery | NICE 2016 | Very low | Weak for |  |
| 1. **Sickle cell disease/trait test** | | | |  |
| Routinely | NICE 2016 | Very low | Weak against |  |
| Assess personal of family history of sickle cell anemia | NICE 2016 | Very low | Weak against |  |
| Contact a specialized service providing treatment to a confirmed case | NICE 2016 | Very low | Weak for |  |
| 1. **Chest X-ray** | | | |  |
| Routinely in healthy people | NICE 2016; SARNEPI 2014;  SAC 2016 | Low | Strong against |  |
| Patients with a history or diagnostic tests suggesting cardiorespiratory disease | SBC 2017 | Low | Strong for | PY |
|  | SARNEPI 2014 | Low | Weak for |  |
|  | SAC 2016 | Moderate |  |  |
| Patients over 40 years, patients undergoing non-low-risk surgery | SBC 2017 | Low | Weak for |  |
| Patients undergoing non-low-risk surgery or mainly intrathoracic or intraabdominal surgery | SBC 2017 | Low | Weak for |  |
|  | SAC 2016 | Moderate |  |  |
| 1. **Prokinetic and other interventions** | | | |  |
| Routine use of antacids, metoclopramide or H2-receptor antagonists before elective surgery in non-obstetric patients | ESA 2011 | High | Strong against |  |
| H2-receptor antagonists the night before and the morning of elective caesarean section | ESA 2011 | Moderate | Strong for |  |
| Intravenous H2-receptor antagonist before emergency caesarean section; supplemented with 30 ml of sodium citrate if general anesthesia is planned | ESA 2011 | Moderate | Strong for |  |
| 1. **Troponin** | | | |  |
| Troponin prior to vascular surgery | SBC 2017 | Moderate | Weak for |  |
| Troponin as a preoperative marker of cardiovascular risk and mortality in non-cardiac surgery | SBC 2017 | Low | Weak for |  |
| 1. **Stress Testing** | | | |  |
| In high-risk patients with unknown functional capacity | ACC/AHA 2014 | Moderate | Weak against | ? |
|  | SAC 2016 | High | Weak for |  |
| Patients with major criteria of high cardiovascular risk (**eTable 5**) | SAC 2016 | Low | Strong against |  |
| For high-risk patients and moderate to good (≥4 METs to 10 METs) functional capacity | ACC/AHA 2014 | Low | Weak against |  |
| For high-risk patients and poor (<4 METs) or unknown functional capacity, if it will change management. | ACC/AHA 2014 | Low | Weak against |  |
| Patients with low risk and a poor (<4METs) or unknown functional capacity, who have angina or dyspnea functional class I-II | SAC 2016 | Low | Weak for |  |
| Patients with low clinical risk criteria established in **eTable 5**, who are asymptomatic and with good functional class | SAC 2016 | Low | Weak against |  |
| Routinely for patients undergoing low-risk noncardiac surgery | ACC/AHA 2014 | Moderate | Strong against |  |
|  | CCSG 2017 | Very low |  |  |
| 1. **Stress test image** | | | |  |
| For high-risk surgery patients with two or more clinical risk factors and low functional capacity | ESC/ESA 2014 | Low | Strong for |  |
| For intermediate and high-risk patients with one or two clinical risk factors and poor functional capacity (<4MET) | ESC/ESA 2014 | Very low | Weak against |  |
| For low-risk patients regardless of the clinic state patient | ESC/ESA 2014 | Very low | Strong against |  |
|  | CCSG 2017 | Low |  |  |
| 1. **Noninvasive test for myocardial ischemia** | | | |  |
| Patients undergoing intermediate or high-risk surgery (without severe cardiovascular perioperative conditions) and those undergoing arterial vascular surgery | SBC 2017 | Moderate | Weak for |  |
| Intermediate or high-risk patients with poor functional capacity undergoing intermediate-risk surgery | SBC 2017 | Moderate | Weak against |  |
| Patients undergoing low-risk surgery | SBC 2017 | Low | Strong against |  |
| Low-risk patients undergoing low or intermediate-risk surgery | SBC 2017 | Low | Strong against |  |
| 1. **Cardiopulmonary Stress Test** | | | |  |
| Cardiopulmonary exercise testing to improve the estimation of cardiac risk | CCSG 2017 | Low | Strong against |  |
| High-risk patients with unknown functional capacity | ACC/AHA 2014 | Moderate | Weak against |  |
| 1. **Pharmacological stress test** | | | |  |
| Patients undergoing non-cardiac surgery who have poor functional capacity (<4 METS) [dobutamine stress test] | ACC/AHA 2014 | Moderate | Weak for |  |
| Routinely in asymptomatic patients who are at low-risk surgery | ACC/AHA 2014 | Moderate | Strong against |  |
| 1. **Pregnancy testing** | | | |  |
| Performed in women of childbearing age | SARNEPI 2014 | Very low | Weak for |  |
| - Test the day of surgery in women of childbearing age. - In pregnant women, ensure that surgery and anesthesia does not threaten the fetus life. - Document all discussions with women about whether to carry out a pregnancy test. - Carry out the pregnancy test under the possibility of pregnancy. | NICE 2016 | Very low | Strong for |  |
| 1. **coronary angiography** | | | |  |
| The indications of angiography and coronary revascularization are those of non-surgical context | ESC/ESA 2014 | Moderate | Strong for |  |
| Urgent angiography in patients with myocardial infarction without ST elevation requiring elective non-cardiac surgery or with a computed tomography (CT) with multiple cuts showing serious injury of the left coronary trunk | ESC/ESA 2014 | High | Strong for | Y |
|  | SBC 2017 | Moderate |  |  |
|  | SAC 2016 | Low | Weak for |  |
| Urgent or early invasive strategy for patients with NSTEMI requiring elective non-cardiac surgery | ESC/ESA 2014 | Moderate | Strong for |  |
|  | SBC 2017 | High |  |  |
| Patients with recent coronary disease at high clinical risk, functional class III-IV in the last 6 months, or patients with severe valve disease and concomitant coronary heart disease | SAC 2016 | Low | Strong for |  |
| Patients with non-high-risk criteria (**eTable 5**) and functional or pharmacological stress tests showing myocardial ischemia | SAC 2016 | Low | Weak against |  |
| Patients with or without stable coronary disease functional class I-II without evidence of ischemia by stress tests, or those with severe coronary disease according CT multislice (excluding injury of left coronary trunk) clinically stable without ischemia, or in patients whose non-cardiac surgery cannot be delayed more than two weeks due to the underlying disease | SAC 2016 | Low | Strong against |  |

***** See Direction and strength recommendation rule to do or not to do discrepant interventions in Guideline quality appraisal and classification section. **Y**: Yes, **N**: No do it, **PY**: Probably yes, **PN**: Probably No do it, **?:** Uncertainty to do it.

**Doing or not doing the recommendation rule:**

**Y** or **N** do it: ≥2/3 recommendations in the same direction (for/against) and ≥2/3 strong recommendations.

**PY** or **PN** do it: ≥2/3 recommendations in the same direction (for/against) and <2/3 strong recommendations.

**Uncertainty** **(?)** to do it: <2/3 recommendations in the same direction (for/against).

**Table 2. Recommended risk stratification evaluations only**

| **Recommendation** | **Guides** | **Do?*** |
| --- | --- | --- |
| **1. Electrocardiography:** | | |
| People with a morbidity undergoing intermediate or major surgery | ESC/ESA 2014; SAC 2016; SBC 2017 | Y |
| In neonates and/or children of 6 months | SARNEPI 2014 | PY |
| Perform in cases of clinical suspicion | SARNEPI 2014 | PY |
| People with cardiovascular disease | ACC/AHA 2014; NICE 2016; SAC 2016 (­↑); SBC 2017 (↑↑­­) | PY |
| **2. Effort electrocardiography** | | |
| Patients undergoing surgeries of intermediate or high risk of complications, including arterial vascular surgery (without severe cardiovascular perioperative conditions) | SBC 2017 | PY |
| **3. Resting echocardiography** | | |
| **High-risk surgery** | | |
| Patient with suspected moderate or significant valvular involvement without evaluation in the last year or with worsening of symptoms | SBC 2017 | Y |
| Patient with heart failure or symptoms suggestive heart problems with cardiac surgical indication and without assessment in the past year | SBC 2017; SAC 2016 | PY |
| Symptomatic patients with stent grafts who go to surgery and who have no evaluation in the last year | SBC 2017; SAC 2016 | PY |
| Asymptomatic patients | SBC 2017 | PY |
| **5. Complete laboratory** | | |
| Patients undergoing high-risk surgery | NICE 2016 | Y |
| Patients with renal or cardiovascular disease undergoing intermediate-risk surgery that has not been recently evaluated | NICE 2016 | PY |
| Patients with preeclampsia or other preceding or a suspect of hemostatic disorder, it is recommended to apply platelet count, liver function test and evaluation of coagulation | BARA 2013 | PY |
| In case of bleeding or complications history of previous alloimmunization, it is recommended to evaluate the blood type. | BARA 2013 | PY |
| **7. Urinalysis** | | |
| Urine or culture if diagnosing an urinary infection can influence surgery decisions | NICE 2016 | PY |
| **8. Hemostasis/Coagulation Tests** | | |
| Patients with a history of bleeding | SBC 2017 | Y |
| Patients with liver failure | SBC 2017; NICE 2016 | Y |
| Anticoagulated patients (e.g., consume Warfarin) | SBC 2017; NICE 2016 | Y |
| Patients with potential risk of bleeding undergoing intermediate or high-risk surgery | SBC 2017; ICSI 2012; NICE 2016 (­­↑↑); SARNEPI 2014 (↑­) | Y |
| **9. Hematocrit and hemoglobin** | | |
| In pediatric patients with possible bleeding | SARNEPI 2014 | Y |
| Patients with anemia or blood disease or liver disease; when you suspected of anemia or other chronic disease during clinical examination. In medium or high-risk surgeries anticipated transfusion requirement | SBC 2017/ICSI 2012 | Y |
| Patients requiring intermediate or major surgery, and bleeding risk of transfusion requirement | SBC 2017 | Y |
| Patient s with a history of hematological or liver disease | SBC 2017 | Y |
| Patients over 40 years | SBC 2017 | PY |
| **10. Glycated hemoglobin (HbA1c) test** | | |
| Diabetic patient without Hb1Ac within 3 months | NICE 2016 | PY |
| **11. Polysomnography** | | |
| In patients requiring continuous positive airway pressure (CPAP) | ICSI 2012 | Y |
| **12. Preoperative evaluation** | | |
| It is carried off in all pediatric patients receiving anesthesia | SARNEPI 2014 | Y |
| All patients who undergoing diagnostic or therapeutic procedures | ICSI 2012; BARA 2013 | PY |
| Patients with ASA 1 or 2 without surgical or obstetric history (preanesthetic evaluation, including physical examination, the day of the procedure). | BARA 2013 | PY |
| Patient with significant medical, surgical or obstetrical history (anesthesiologist assessment) | BARA 2013 | PY |
| **13. Assessment of left ventricular function** | | |
| Suspected valvular disease with relevant clinical manifestations; Preoperative evaluation of patients who will receive liver transplantation | SBC 2017 (­­); ACC/AHA 2014 (­) | PY |
| Patients undergoing high-risk surgery | SBC 2017 | PY |
| Obese patients (BMI ≥40) undergoing bariatric surgery | SBC 2017 | PY |
| **14. Assessment of risk factors for surgical site infection** | | |
| Assessment of smoking, diabetes, obesity, malnutrition and chronic skin disease | ICSI 2012 | Y |
| **15. Glucose** | | |
| Diabetic patients | ICSI 2012 | Y |
| **16. Informed consent (Ideally written)** | | |
| Provide information on risks and benefits related to obstetric anesthesia and analgesia. | BARA 2013 | PY |
| **17. Natriuretic peptide** | | |
| Patients undergoing vascular surgery | SBC 2017 | PY |
| Patients over 55 years with at least one cardiovascular risk factor undergoing non-cardiovascular surgery | SBC 2017 | PY |
| **18. Brain natriuretic peptide (BNP) or NT-proBNP** | | |
| Patients over 65 years or patients between 45 and 64 years with significant cardiovascular disease or score (revised cardiac risk index (RCRI) ≥ 1 | CCSG 2017 | Y |
| **19. Pulmonary function tests** | | |
| Assessment by medical senior anesthesiologist after confirming respiratory illness or suspected in patients ASA 3 or 4 undergoing high-risk surgery | NICE 2016 | PY |
| **20. Kidney function tests** | | |
| For complex or major surgery in ASA 1 patients at risk of acute kidney injury (AKI) | NICE 2017 | PY |
| In intermediate-risk surgery in ASA 2 patients at risk of AKI. In patients with increased risk surgery performed | NICE 2016 | PY |
| Low-risk surgery in ASA 3/4 patients at risk of AKI or higher-risk surgery in ASA 3/4 patients | NICE 2016 | PY |
| **21. Sickle cell disease/trait test** | | |
| Contact a specialized service providing treatment to a confirmed case | NICE 2016 | PY |
| **22. Chest X-ray** | | |
| Patients with a history or diagnostic test suggests cardiorespiratory disease | SBC 2017 (↑↑­­); SARNEPI 2014; SAC 2016 (↑­) | PY |
| Patients over 40 years, patients undergoing non-low-risk surgery | SBC 2017 | PY |
| Patients undergoing non-low-risk surgery or mainly intrathoracic or intraabdominal surgery | SBC 2017; SAC 2016 | PY |
| **23. Prokinetic and other interventions** | | |
| H2-receptor antagonists the night before and the morning of elective caesarean section | ESA 2011 | Y |
| Intravenous H2-receptor antagonist before emergency caesarean section; supplemented with 30 ml of sodium citrate 0.3mm/L if general anesthesia is planned | ESA 2011 | Y |
| **24. Troponin** | | |
| Troponin prior to vascular surgery | SBC 2017 | PY |
| Troponin as a preoperative marker of cardiovascular risk and mortality in non-cardiac surgery | SBC 2017 | PY |
| **25. Stress Testing** | | |
| Patients with low risk and a poor (<4METs) or unknown functional capacity, who have angina or dyspnea functional class I-II | SAC 2016 | PY |
| **26. Stress test image** | | |
| For high-risk surgery patients with two or more clinical risk factors and low functional capacity | ESC/ESA 2014 | Y |
| **27. Noninvasive test for myocardial ischemia** | | |
| Patients undergoing intermediate or high-risk surgery (without severe cardiovascular perioperative conditions) and those undergoing arterial vascular surgery | SBC 2017 | PY |
| **29. Pharmacological stress test** | | |
| Patients undergoing non-cardiac surgery who have poor functional capacity (<4 METS) [dobutamine stress test] | ACC/AHA 2014 | PY |
| **30. Pregnancy testing** | | |
| Test the day of surgery in women of childbearing age. In pregnant women, ensure that surgery and anesthesia does not threaten the fetus life. Document all discussions with women about whether to carry out a pregnancy test. Carry out the pregnancy test under the possibility of pregnancy. | NICE 2016 | Y |
| Performed in women of childbearing age | SARNEPI 2014 | PY |
| **31. coronary angiography** | | |
| The indications of angiography and coronary revascularization are those of non-surgical context | ESC/ESA 2014 | Y |
| Urgent angiography in patients with myocardial infarction without ST elevation requiring elective non-cardiac surgery or with a computed tomography (CT) with multiple cuts showing serious injury of the left coronary trunk | ESC/ESA 2014; SBC 2017 (­­↑↑); SAC 2016 (­↑) | Y |
| Urgent or early invasive strategy for patients with NSTEMI requiring elective non-cardiac surgery | ESC/ESA 2014; SBC 2017 | Y |
| Patients with recent coronary disease at high clinical risk, functional class III-IV in the last 6 months, or patients with severe valve disease and concomitant coronary heart disease | SAC 2016 | Y |

***** See Direction and strength recommendation rule to do or not to do discrepant interventions in Guideline quality appraisal and classification section. **Y**: Yes, **N**: No do it, **PY**: Probably yes, **PN**: Probably No do it, **?:** Uncertainty to do it.

**Strong** **for** (**↑↑), Strong** **against** (**↓↓)**, **Weak** **for** (**↑)**, **Weak** **against** (**↓)**

**Table 3. Not recommended risk stratification evaluations**

| **Recommendation** | **Guides** | **Do?** |
| --- | --- | --- |
| **1. Electrocardiography:** | | |
| Healthy people undergoing minor surgery | NICE 2016; SBC 2017; SARNEPI 2014; ESC/ESA 2014; ICSI 2012 (↓↓); SAC 2016 (↓) | N |
| **2. Effort electrocardiography** | | |
| Patients undergoing low risk surgery | SBC 2017 | N |
| Patients undergoing intermediate risk surgery | SBC 2017 | N |
| **3. Resting echocardiography** | | |
| **Low, intermediate or uncertain surgical risk** | | |
| Routine test in asymptomatic patients without suspect of heart failure or severe valvular disease | SBC 2017; NICE 2016; CCSG 2017 (↓); SAC 2016 (↓↓) | PN |
| **4. Effort echocardiography** | | |
| Routinely to assess cardiac risk | CCSG 2017 | N |
| **5. Complete laboratory** | | |
| Patients undergoing low-risk surgery independently of their ASA score | NICE 2016 | N |
| Patients undergoing intermediate-risk surgery | NICE 2016 | N |
| **6. Tomographic coronary angiography** | | |
| Routinely to assess cardiac risk | CCSG 2017 | N |
| **7. Urinalysis** | | |
| Routinely before surgery | NICE 2016 | PN |
| **8. Hemostasis/Coagulation Tests** | | |
| Pediatric patients with negative history | SARNEPI 2014 | N |
| Routinely before surgery | NICE 2016 | N |
| **9. Hematocrit and hemoglobin** | | |
| In pediatric patients for minor surgery | SARNEPI 2014 | N |
| **10. Glycated hemoglobin (HbA1c) test** | | |
| Patients without diabetes | NICE 2016 | PN |
| **11. Polysomnography** | | |
| **12. Preoperative evaluation** | | |
| Emergency surgeries in pediatric patients | SARNEPI 2014 | N |
| **13. Assessment of left ventricular function** | | |
| Routinely | ACC/AHA 2014 | N |
| **15. Glucose** | | |
| Routinely to pediatric patients | SARNEPI 2014 | N |
| **19. Pulmonary function tests** | | |
| Spirometry in patients undergoing non-high-risk surgery | NICE 2016 | N |
| Arterial blood gas analysis in patients undergoing non-high-risk surgery | NICE 2016 | N |
| **20. Kidney function tests** | | |
| For minor surgery in ASA 1/2 patients or intermediate-risk surgery in ASA 2 patients | NICE 2016 | PN |
| **21. Sickle cell disease/trait test** | | |
| Routinely | NICE 2016 | PN |
| Assess personal of family history of sickle cell anemia | NICE 2016 | PN |
| **22. Chest X-ray** | | |
| Routinely in healthy people | NICE 2016; SARNEPI 2014; SAC 2016 | N |
| **23. Prokinetic and other interventions** | | |
| Routine use of antacids, metoclopramide or H2-receptor antagonists before elective surgery in non-obstetric patients | ESA 2011 | N |
| **25. Stress Testing** | | |
| For high-risk patients and moderate to good (≥4 METs to 10 METs) functional capacity | ACC/AHA 2014 | PN |
| For high-risk patients and poor (<4 METs) or unknown functional capacity, if it will change management. | ACC/AHA 2014 | PN |
| Patients with low clinical risk criteria established in **Annex 5**, who are asymptomatic and with good functional class | SAC 2016 | PN |
| Patients with major criteria of high cardiovascular risk (**Annex 5**) | SAC 2016 | N |
| Routinely for patients undergoing low-risk noncardiac surgery | ACC/AHA 2014; CCSG 2017 | N |
| **26. Stress test image** | | |
| For intermediate and high-risk patients with one or two clinical risk factors and poor functional capacity (<4MET) | ESC/ESA 2014 | PN |
| For low-risk patients regardless of the clinic state patient | ESC/ESA 2014; CCSG 2017 | N |
| **27. Noninvasive test for myocardial ischemia** | | |
| Intermediate or high-risk patients with poor functional capacity undergoing intermediate-risk surgery | SBC 2017 | PN |
| Patients undergoing low-risk surgery | SBC 2017 | N |
| Low-risk patients  undergoing low or intermediate-risk surgery | SBC 2017 | N |
| **28. Cardiopulmonary Stress Test** | | |
| High-risk patients with unknown functional capacity | ACC/AHA 2014 | PN |
| Cardiopulmonary exercise testing to improve the estimation of cardiac risk | CCSG 2017 | N |
| **29. Pharmacological stress test** | | |
| Patients undergoing non-cardiac surgery who have poor functional capacity (<4 METS) [dobutamine stress test] | ACC/AHA 2014 | PY |
| Routinely in asymptomatic patients who are at low-risk surgery | ACC/AHA 2014 | N |
| **31. coronary angiography** | | |
| Patients with non-high-risk criteria (**Annex 5**) and functional or pharmacological stress tests showing myocardial ischemia | SAC 2016 | PN |
| Patients with or without stable coronary disease functional class I-II without evidence of ischemia by stress tests, or those with severe coronary disease according CT multislice (excluding injury of left coronary trunk) clinically stable without ischemia, or in patients whose non-cardiac surgery cannot be delayed more than two weeks due to the underlying disease | SAC 2016 | N |

***** See Direction and strength recommendation rule to do or not to do discrepant interventions in Guideline quality appraisal and classification section. **Y**: Yes, **N**: No do it, **PY**: Probably yes, **PN**: Probably No do it, **?:** Uncertainty to do it.

**Strong** **for** (**↑↑), Strong** **against** (**↓↓)**, **Weak** **for** (**↑)**, **Weak** **against** (**↓)**

**Appendix 7**

**Table 1. Specific antibiotic recommendations for each surgical site**

| **Clinical Practice Guideline** | **Type of Surgery** | **Antibiotic recommendation** | **Strength of Recommendation** |
| --- | --- | --- | --- |
| ***ASHP 2013*** | Heart | Cefazolin, cefuroxime | Strong |
|  | Chest | Cefazolin, ampicillin-sulbactam | Strong |
|  | gastroduodenal | cefazolin | Strong |
|  | Biliary tract | Cefazolin, cefoxitin, cefotetana, ceftriaxone, ampicillin-sulbactam | Strong |
|  | appendectomy | Cefoxitin, cefotetana, cefazolin + metronidazole | Strong |
|  | Neurosurgery | cefazolin | Strong |
|  | Ophthalmology | Topical agents: neomycin-polymyxin B-gramicidin or 4th generation fluoroquinolones (gatifloxacin or moxifloxacin) one drop every 5 to 15 minutes for 5 doses | Low |
|  | Urology | Fluoroquinolones, trimethoprim-sulfamethoxazole, cefazolin | Strong |
|  | Vascular | cefazolin | Strong |
|  | Liver transplant | Piperacillin-tazobactam, ampicillin cefotaxime + | Low |
|  | Plastic surgery | Cefazolin, ampicillin-sulbactam | Strong |
|  | Pancreas/kidney. | Cefazolin, fluconazole (for patients at high risk for fungal infections) | Strong |
|  | Hysterectomy | Cefazolin, cefotetan, cefoxitin, ampicillin-sulbactam | Strong |
|  | Caesarean section. | cefazolin | Strong |

## **Appedix 8**

## **Table 1.** **Therapeutic/preventive care, GRADE level of evidence and strength of recommendation**

| **Recommendation** | **Guide** | **Level of Evidence** | **Strength of Recommendation** | **Do*** |
| --- | --- | --- | --- | --- |
| 1. **Smoking cessation** | | | |  |
| Smoking cessation advice | ERAS 2012,  ESC/ESA 2014 | Low | Strong for |  |
| 1. **Fast** | | | |  |
| Stop fluid intake in children and adults at least 2 hours before elective surgery in | ESA 2011 | High | Strong for |  |
|  | ERAS 2012 | Moderate |  |  |
| Stop intake of solids in children and adults 6 hours before surgery | ESA 2011 | Moderate |  |  |
| Stop intake in infants up to 4 hours before surgery and 6 hours in those who consume other milks | ESA 2011 | Low |  |  |
| Intake of clear fluids (including water, clear juice and tea or coffee without milk) in children and adults up to 2 hours before elective surgery. | ESA 2011 | Moderate |  |  |
| 1. **Carbohydrate intake** | | | |  |
| Intake until 2 hours before surgery in nondiabetics | ESA 2011; ERAS 2012 | Moderate | Strong for |  |
| Taking high carbohydrate drinks to 2 hours before elective surgery even in diabetic patients | ESA 2011 | High |  |  |
| Drink liquids rich in carbohydrates before elective surgery improves subjective well-being, reduces thirst and hunger and reduces postoperative insulin resistance | ESA 2011 | High |  |  |
| 1. **Alcohol intake** | | | |  |
| Avoid drinking 4 weeks before, especially in rectal surgery. | ERAS 2012;  ERAS 2016 | Moderate | Strong for |  |
| 1. **Bowel preparation (cleansing)** | | | |  |
| With or without planned bowel resection | ERAS 2016 | Moderate | Strong against |  |
| 1. **Antimicrobial prophylaxis** (See **eTable 2** for specific antibiotic recommendations details) | | | |  |
| Antibiotics intravenous (first generation cephalosporin or amoxicillin/clavulanate) routinely 60 minutes before the incision. Further doses for prolonged surgery, severe blood losses and obese patients | ERAS 2016;  ERAS 2012;  PNLG 2009 | High | Strong for | Y |
|  | STS 2007;  CDC 2017 | Moderate |  |  |
|  | SIGN 2014 | Low | Weak for |  |
| Vancomycin monotherapy | STS 2007 | Low | Weak against |  |
| For insertion of a pacemaker or cardiac defibrillator, in open surgery including coronary bypass and valve prosthesis placement | PNLG 2009 | High | Strong for |  |
| For lung resection | PNLG 2009 | Moderate | Strong for |  |
| For clean-contaminated head and neck surgery | PNLG 2009 | High | Strong for |  |
| For adenotonsillectomy | PNLG 2009 | High | Weak against |  |
| For ear surgery including myringoplasty | PNLG 2009 | High | Strong against |  |
| For nasal and paranasal surgeries sinus | PNLG 2009 | Moderate | Strong against |  |
| For clean head and neck surgery | PNLG 2009 | Very low | Strong against |  |
| For colorectal surgery | PNLG 2009 | High | Strong for |  |
| For oncological breast surgery and reduction mammoplasty | PNLG 2009 | High | Strong for |  |
| For endoscopic gastrostomy and stomach and duodenum surgery | PNLG 2009 | Moderate | Strong for |  |
| For clean-contaminated procedures esophagus and small intestine | PNLG 2009 | Very low | Weak for |  |
| For appendectomy, open biliary surgery, liver resection surgery, pancreatic surgery, breast augmentation | PNLG 2009 | High | Strong for |  |
| For inguinal hernia repair with or without use of prosthetic material, laparoscopic hernia surgery with or without prosthetic material, diagnostic laparoscopy and excisional lymph node biopsy | PNLG 2009 | High | Strong against |  |
| For laparoscopic cholecystectomy surgery | PNLG 2009 | High | Strong against |  |
| Intranasal mupirucina in adult patients undergoing surgery with a high risk of major morbidity due to S. aureus or MRSA | SIGN 2014 | High | Strong for |  |
| For craniotomy and cerebrospinal flow deviation | PNLG 2009 | High | Strong for |  |
| For induction of abortion and caesarean section | PNLG 2009 | High | Strong for |  |
|  | SIGN 2014 |  |  |  |
|  | CDC 2017 |  |  |  |
| For abdominal and vaginal hysterectomy | PNLG 2009 | Moderate | Strong for |  |
| For salpingo-oophorectomy and ovarian tissue excision or reconstruction | PNLG 2009 | High | Strong against |  |
| For ankle prosthesis implantation | PNLG 2009 | High | Strong for |  |
| For knee prosthesis implantation | PNLG 2009 | Low | Strong for |  |
| For closed fracture fixation, mounting a prosthetic device when there is no direct evidence available, ankle fracture repair | PNLG 2009 | High | Strong for |  |
| For spinal surgery | PNLG 2009 | Moderate | Strong for |  |
| For elective orthopedic surgeries without use of prosthesis | PNLG 2009 | Very low | Strong against |  |
| For transurethral resection of the prostate, lithotripsy | PNLG 2009 | High | Strong for |  |
| For transrectal prostate biopsy, radical prostatectomy, radical cystectomy, surgery of renal parenchyma, nephrectomy and removal of hydrocele | PNLG 2009 | Moderate | Strong for |  |
| For transurethral resection of bladder tumors | PNLG 2009 | Very low | Strong against |  |
| For lower limb amputation and arterial surgery in the abdomen or lower extremities | PNLG 2009 | Moderate | Strong for |  |
| For carotidal thrombo-endarterectomy, endarterectomy, tubal surgery varicose veins and other venous occlusions | PNLG 2009 | Very low | Strong against |  |
| Antibiotic must have a spectrum of action against likely contaminants | PNLG 2009 | Very low | Weak for |  |
| Avoid beta-lactam antibiotics in patients with a history of anaphylaxis, urticaria or rash appearing immediately after treatment with penicillin | PNLG 2009 | Very low | Weak for |  |
|  | SIGN 2014 | Low |  |  |
| Antibiotic prophylaxis should begin immediately before anesthesia and, in any case, of 30 to 60 minutes before the first skin incision | PNLG 2009 | Moderate | Strong for |  |
|  | SIGN 2014 | High |  |  |
| More than single antibiotic dose (except in special situations) | SIGN 2014 | Very low | Strong against |  |
| Additional intraoperative dose of antibiotic in adults, to be held after the fluid replenishment, if a loss of more than 1500 ml of blood is verified during the operation or after hemodilution of more 15 ml per kg | PNLG 2009 | Very low | Weak for |  |
| Consider the increased risk clostridium difficile infection associated with some antibiotics like cephalosporins, clindamycin, fluoroquinolones, carbapenems | SIGN 2014 | Low | Weak for |  |
| Consider glycopeptides for prophylaxis in patients undergoing high-risk surgery that are positive for MRSA | SIGN 2014 | High | Strong for |  |
| Registering a minimum set of data on medical history and treatment forms to assess the suitability of perioperative antibiotic prophylaxis | PNLG 2009 | Very low | Strong for |  |
| 1. **Preanesthetic medication** | | | |  |
| Benzodiazepines | ERAS 2012; ERAS 2015 | Moderate | Weak against |  |
| 1. **Thromboprophylaxis** | | | |  |
| Compression stockings | ERAS 2012 | High | Strong for |  |
| Low molecular weight heparin | ERAS 2012; ERAS 2016 |  |  |  |
| Continuation of contraceptives | ERAS 2016 |  |  |  |
| 1. **Surgical site preparation** | | | |  |
| Alcohol-chlorhexidine use | ERAS 2012;  ERAS 2016;  CDC 2017 | High | Strong for |  |
| Antimicrobial agents (i.e. ointments, solutions or powders) for prevention of surgical site infection | CDC 2017 | Low | Strong against |  |
| Hair clipping | ERAS 2016 | High | Strong for |  |
| Adhesive strips of plastic with or without antimicrobial properties | CDC 2017 | Moderate | Weak against |  |
| Microbial sealant after intraoperative skin preparation | CDC 2017 | Low | Weak against |  |
| Patients bath with antiseptic agent at least one night before surgery | CDC 2017 | Moderate | Strong for |  |
| 1. **Adjustments of insulin therapy in diabetic patients** | | | |  |
| 50% reduction in long-acting insulin | ICSI 2012 | Low | Strong for |  |
| Correction with short-acting insulin | ICSI 2012 | Low | Strong for |  |
| Oral hypoglycemic agents | ICSI 2012 | Low | Strong for |  |
| 1. **Beta-blockers** | | | |  |
| Continuation of beta-blockers | ICSI 2012 | Low | Strong for | PY |
|  | SAC 2016 | Low | Weak for |  |
| For patients with positive test for myocardial ischemia undergoing vascular surgery | ICSI 2012 | Low | Strong for | PY |
|  | SAC 2016 | Low | Weak for |  |
| Start the day of surgery treatment regardless of the condition to be treated | SAC 2016 | High | Strong against |  |
| 1. **Prokinetic** | | | |  |
| For obstetrical patients | ERAS 2011 | Moderate | Strong for |  |
| For non-obstetrical patients | ERAS 2011 | Moderate | Strong against |  |
| 1. **Statins** | | | |  |
| Continuation of statins or start before undergoing noncardiac surgery patients with significant atherosclerosis as secondary prevention | SAC 2016 | Low | Weak for |  |
| Treatment naïve patients undergoing noncardiac surgery without significant atherosclerosis | SAC 2016 | Low | Strong against |  |
| 1. **Aspirin** | | | |  |
| Suspending aspirin three or more days before noncardiac surgery and not restart within a week after it | SAC 2016 | High | Strong for |  |
| Continuation of aspirin (75-100 mg daily) in patients who presented acute coronary syndrome in the last 12 months or history of percutaneous coronary intervention | SAC 2016 | Low | Weak for |  |
| Start or not to suspend treatment prior to surgery | SAC 2016 | High | Strong against |  |
| 1. **Renin-angiotensin system inhibitors** | | | |  |
| Suspend them the day of surgery in chronically medicated patients and restart immediately in hemodynamically stable conditions | SAC 2016 | Low | Weak for |  |
| Start in patients with severe hypertension or ventricular dysfunction if suspending the day of surgery |  |  |  |  |
| Start treatment the day of surgery in patients who do not receive it chronically | SAC 2016 | Low | Strong against |  |
| 1. **Calcium Channel Blockers** | | | |  |
| Suspend only the preoperative dose the day of the surgery in chronically medicated patients | SAC 2016 | Low | Weak for |  |
| Starting treatment in patients with inducible myocardial ischemia or suspected coronary vasospasm during preoperative evaluation and suspend only the day of surgery |  |  |  |  |
| Starting calcium channel blockers in the preoperative surgery in patients who do not receive chronically | SAC 2016 | Low | Strong against |  |

***** See Direction and strength recommendation rule to do or not to do discrepant interventions in Guideline quality appraisal and classification section. **Y**: Yes, **N**: No do it, **PY**: Probably yes, **PN**: Probably No do it, **?:** Uncertainty to do it.

**Doing or not doing the recommendation rule:**

**Y** or **N** do it: ≥2/3 recommendations in the same direction (for/against) and ≥2/3 strong recommendations.

**PY** or **PN** do it: ≥2/3 recommendations in the same direction (for/against) and <2/3 strong recommendations.

**Uncertainty** **(?)** to do it: <2/3 recommendations in the same direction (for/against).

## **Table 2. Recommended therapeutic/preventive care**

| **Recommendation** | **Guide** | **Do?** |
| --- | --- | --- |
| **1.    Smoking cessation** | | |
| Smoking cessation advice | ERAS 2012, ESC/ESA 2014 | Y |
| **2.    Fast** | | |
| Stop fluid intake in children and adults at least 2 hours before elective surgery in | ESA 2011; ERAS 2012 | Y |
| Stop intake of solids in children and adults 6 hours before surgery | ESA 2011 | Y |
| Stop intake in infants up to 4 hours before surgery and 6 hours in those who consume other milks | ESA 2011 | Y |
| Intake of clear fluids (including water, clear juice and tea or coffee without milk) in children and adults up to 2 hours before elective surgery. | ESA 2011 | Y |
| **3.    Carbohydrate intake** | | |
| Intake until 2 hours before surgery in nondiabetics | ESA 2011; ERAS 2012 | Y |
| Taking high carbohydrate drinks to 2 hours before elective surgery even in diabetic patients | ESA 2011 | Y |
| Drink liquids rich in carbohydrates before elective surgery improves subjective well-being, reduces thirst and hunger and reduces postoperative insulin resistance | ESA 2011 | Y |
| **4.    Alcohol intake** | | |
| Avoid drinking 4 weeks before, especially in rectal surgery. | ERAS 2012; ERAS 2016 | Y |
| **5.    Antimicrobial prophylaxis** (See **Annex 2** for specific antibiotic recommendations details) | | |
| Antibiotics intravenous (first generation cephalosporin or amoxicillin/clavulanate) routinely 60 minutes before the incision. Further doses for prolonged surgery, severe blood losses and obese patients | ERAS 2012; PNLG 2009; STS 2007; CDC 2017 (↑↑); SIGN 2014 (↑) | Y |
| For insertion of a pacemaker or cardiac defibrillator, in open surgery including coronary bypass and valve prosthesis placement | PNLG 2009 | Y |
| For lung resection | PNLG 2009 | Y |
| For clean-contaminated head and neck surgery | PNLG 2009 | Y |
| For colorectal surgery | PNLG 2009 | Y |
| For oncological breast surgery and reduction mammoplasty | PNLG 2009 | Y |
| For endoscopic gastrostomy and stomach and duodenum surgery | PNLG 2009 | Y |
| For appendectomy, open biliary surgery, liver resection surgery, pancreatic surgery, breast augmentation | PNLG 2009 | Y |
| Intranasal mupirucina in adult patients undergoing surgery with a high risk of major morbidity due to S. aureus or MRSA | SIGN 2014 | Y |
| For craniotomy and cerebrospinal flow deviation | PNLG 2009 | Y |
| For induction of abortion and caesarean section | PNLG 2009; SIGN 2014; CDC 2017 | Y |
| For abdominal and vaginal hysterectomy | PNLG 2009 | Y |
| For ankle prosthesis implantation | PNLG 2009 | Y |
| For knee prosthesis implantation | PNLG 2009 | Y |
| For closed fracture fixation, mounting a prosthetic device when there is no direct evidence available, ankle fracture repair | PNLG 2009 | Y |
| For spinal surgery | PNLG 2009 | Y |
| For transurethral resection of the prostate, lithotripsy | PNLG 2009 | Y |
| For transrectal prostate biopsy, radical prostatectomy, radical cystectomy, surgery of renal parenchyma, nephrectomy and removal of hydrocele | PNLG 2009 | Y |
| For lower limb amputation and arterial surgery in the abdomen or lower extremities | PNLG 2009 | Y |
| Antibiotic prophylaxis should begin immediately before anesthesia and, in any case, of 30 to 60 minutes before the first skin incision | PNLG 2009; SIGN 2014 | Y |
| More than single antibiotic dose (except in special situations) | SIGN 2014 | Y |
| Consider glycopeptides for prophylaxis in patients undergoing high-risk surgery that are positive for MRSA | SIGN 2014 | Y |
| Registering a minimum set of data on medical history and treatment forms to assess the suitability of perioperative antibiotic prophylaxis | PNLG 2009 | Y |
| Antibiotic must have a spectrum of action against likely contaminants | PNLG 2009 | PY |
| Avoid beta-lactam antibiotics in patients with a history of anaphylaxis, urticaria or rash appearing immediately after treatment with penicillin | PNLG 2009; SIGN 2014 | PY |
| Additional intraoperative dose of antibiotic in adults, to be held after the fluid replenishment, if a loss of more than 1500 ml of blood is verified during the operation or after hemodilution of more 15 ml per kg | PNLG 2009 | PY |
| Consider the increased risk clostridium difficile infection associated with some antibiotics like cephalosporins, clindamycin, fluoroquinolones, carbapenems | SIGN 2014 | PY |
| **6.    Thromboprophylaxis** | | |
| Compression stockings | ERAS 2012 | Y |
| Low molecular weight heparin | ERAS 2012; ERAS 2016 | Y |
| Continuation of contraceptives | ERAS 2016 | Y |
| **7.    Surgical site preparation** | | |
| Alcohol-chlorhexidine use | ERAS 2012; ERAS 2016; CDC 2017 | Y |
| Hair clipping | ERAS 2016 | Y |
| Patients bath with antiseptic agent at least one night before surgery | CDC 2017 | Y |
| **9. Adjustments of insulin therapy in diabetic patients** | | |
| 50% reduction in long-acting insulin | ICSI 2012 | Y |
| Correction with short-acting insulin | ICSI 2012 | Y |
| Oral hypoglycemic agents | ICSI 2012 | Y |
| **10. Beta-blockers** | | |
| Continuation of beta-blockers | ICSI 2012 (↑↑­­); SAC 2016 (↑­) | PY |
| For patients with positive test for myocardial ischemia undergoing vascular surgery | ICSI 2012 (↑↑­­); SAC 2016 (­↑) | PY |
| **11. Prokinetic** | | |
| For obstetrical patients | ERAS 2011 | Y |
| **12. Statins** | | |
| Continuation of statins or start before undergoing noncardiac surgery patients with significant atherosclerosis as secondary prevention | SAC 2016 | PY |
| **13. Aspirin** | | |
| Suspending aspirin three or more days before noncardiac surgery and not restart within a week after it | SAC 2016 | Y |
| Continuation of aspirin (75-100 mg daily) in patients who presented acute coronary syndrome in the last 12 months or history of percutaneous coronary intervention | SAC 2016 | PY |
| **14. Renin-angiotensin system inhibitors** | | |
| Suspend them the day of surgery in chronically medicated patients and restart immediately in hemodynamically stable conditions | SAC 2016 | PY |
| Start in patients with severe hypertension or ventricular dysfunction if suspending the day of surgery | SAC 2017 | PY |
| **15. Calcium Channel Blockers** | | |
| Suspend only the preoperative dose the day of the surgery in chronically medicated patients | SAC 2016 | PY |
| Starting treatment in patients with inducible myocardial ischemia or suspected coronary vasospasm during preoperative evaluation and suspend only the day of surgery | SAC 2017 | PY |

***** See Direction and strength recommendation rule to do or not to do discrepant interventions in Guideline quality appraisal and classification section. **Y**: Yes, **N**: No do it, **PY**: Probably yes, **PN**: Probably No do it, **?:** Uncertainty to do it.

**Strong** **for** (**↑↑), Strong** **against** (**↓↓)**, **Weak** **for** (**↑)**, **Weak** **against** (**↓)**

## **Table 3. Not recommended therapeutic/preventive care**

| **Recommendation** | **Guide** | **Do?** |
| --- | --- | --- |
| **1. Bowel preparation (cleansing)** | | |
| With or without planned bowel resection | ERAS 2016 | N |
| **2. Antimicrobial prophylaxis** (See **Annex 2** for specific antibiotic recommendations details) | | |
| For ear surgery including myringoplasty | PNLG 2009 | N |
| For nasal and paranasal surgeries sinus | PNLG 2009 | N |
| For clean head and neck surgery | PNLG 2009 | N |
| For inguinal hernia repair with or without use of prosthetic material, laparoscopic hernia surgery with or without prosthetic material, diagnostic laparoscopy and excisional lymph node biopsy | PNLG 2009 | N |
| For laparoscopic cholecystectomy surgery | PNLG 2009 | N |
| For salpingo-oophorectomy and ovarian tissue excision or reconstruction | PNLG 2009 | N |
| For elective orthopedic surgeries without use of prosthesis | PNLG 2009 | N |
| For transurethral resection of bladder tumors | PNLG 2009 | N |
| For carotidal thrombo-endarterectomy, endarterectomy, tubal surgery varicose veins and other venous occlusions | PNLG 2009 | N |
| For adenotonsillectomy | PNLG 2009 | PN |
| For clean-contaminated procedures esophagus and small intestine | PNLG 2009 | PN |
| Vancomycin monotherapy | STS 2007 | PN |
| **3.    Preanesthetic medication** | | |
| Benzodiazepines | ERAS 2012; ERAS 2015 | PN |
| **4.    Surgical site preparation** | | |
| Antimicrobial agents (i.e. ointments, solutions or powders) for prevention of surgical site infection | CDC 2017 | N |
| Adhesive strips of plastic with or without antimicrobial properties | CDC 2017 | PN |
| Microbial sealant after intraoperative skin preparation | CDC 2017 | PN |
| **5. Beta-blockers** | | |
| Start the day of surgery treatment regardless of the condition to be treated | SAC 2016 | N |
| **6. Prokinetic** | | |
| For non-obstetrical patients | ERAS 2011 | N |
| **7. Statins** | | |
| Treatment naïve patients undergoing noncardiac surgery without significant atherosclerosis | SAC 2016 | N |
| **8. Aspirin** | | |
| Start or not to suspend treatment prior to surgery | SAC 2016 | N |
| **9. Renin-angiotensin system inhibitors** | | |
| Start treatment the day of surgery in patients who do not receive it chronically | SAC 2016 | N |
| **10. Calcium Channel Blockers** | | |
| Starting calcium channel blockers in the preoperative surgery in patients who do not receive chronically | SAC 2016 | N |

***** See Direction and strength recommendation rule to do or not to do discrepant interventions in Guideline quality appraisal and classification section. **Y**: Yes, **N**: No do it, **PY**: Probably yes, **PN**: Probably No do it, **?:** Uncertainty to do it.

**Strong** **for** (**↑↑), Strong** **against** (**↓↓)**, **Weak** **for** (**↑)**, **Weak** **against** (**↓)**
